# Transcriptome analysis reveals an Atoh1b-dependent gene set downstream of Dlx3b/4b during early inner ear development in zebrafish

**DOI:** 10.1242/bio.059911

**Published:** 2023-06-05

**Authors:** Diana Ezhkova, Simone Schwarzer, Sandra Spieß, Michaela Geffarth, Anja Machate, Daniela Zöller, Johanna Stucke, Dimitra Alexopoulou, Mathias Lesche, Andreas Dahl, Stefan Hans

**Affiliations:** ^1^ Technische Universität Dresden, Center for Molecular and Cellular Bioengineering (CMCB), CRTD - Center for Regenerative Therapies Dresden, Fetscherstraße 105, 01307 Dresden, Germany; ^2^ Technische Universität Dresden, Center for Molecular and Cellular Bioengineering (CMCB), DRESDEN-Concept Genome Center, Fetscherstraße 105, 01307 Dresden, Germany

**Keywords:** Inner ear, Development, Transcriptome analysis, CRISPR/Cas9, Zebrafish

## Abstract

The vertebrate inner ear is the sensory organ mediating hearing and balance. The entire organ develops from the otic placode, which itself originates from the otic-epibranchial progenitor domain (OEPD). Multiple studies in various species have shown the importance of the forkhead-box and distal-less homeodomain transcription factor families for OEPD and subsequent otic placode formation. However, the transcriptional networks downstream of these factors are only beginning to be understood. Using transcriptome analysis, we here reveal numerous genes regulated by the distal-less homeodomain transcription factors Dlx3b and Dlx4b (Dlx3b/4b). We identify known and novel transcripts displaying widespread OEPD expression in a Dlx3b/4b-dependent manner. Some genes, with a known OEPD expression in other vertebrate species, might be members of a presumptive vertebrate core module required for proper otic development. Moreover, we identify genes controlling early-born sensory hair cell formation as well as regulating biomineral tissue development, both consistent with defective sensory hair cell and otolith formation observed in *dlx3b/4b* mutants. Finally, we show that ectopic Atoh1b expression can rescue early sensorigenesis even in the absence of Dlx3b/4b. Taken together, our data will help to unravel the gene regulatory network underlying early inner ear development and provide insights into the molecular control of vertebrate inner ear formation to restore hearing loss in humans ultimately.

## Teaser

**Summary:** Identification of various new gene sets regulated by the transcription factors Dlx3b/4b during early inner development in zebrafish.

## INTRODUCTION

The vertebrate inner ear is a complex sensory organ mediating hearing and balance through an intricate interplay of mechanosensory hair cells, non-sensory supporting cells and bipolar sensory neurons. Currently, over 5% of the world's population are affected by hearing impairment or deafness and it is estimated that this number rises to over 700 million people by 2050 (www.who.int/news-room/fact-sheets/detail/deafness-and-hearing-loss). Hence, there is a pressing need to develop new therapies restoring hearing abilities and regenerative medicine holds enormous potential. This includes the generation of mechanosensory hair cells and sensory neurons from embryonic stem cells or induced pluripotent stem cells using differentiation protocols that mimic the principles of embryonic inner ear development (Chen and Streit, 2012; Nie and Hashino, 2020; Oshima et al., 2010). However, in order to develop and/or optimize existing protocols, a mechanistic understanding underlying the early events of inner ear development is key.

Inner ear formation is a multistep process initiated with the establishment of the preplacodal region containing the precursors for all sensory placodes (Ladher, 2017; Streit, 2007). Subsequently, the posterior preplacodal region is specified into a common otic-epibranchial progenitor domain (OEPD) that in zebrafish also contains the progenitors of the anterior lateral line ganglion (Chen and Streit, 2012; Hans et al., 2013; McCarroll et al., 2012). Following formation, the otic placode develops into the otocyst or otic vesicle that further acquires the architecture of the adult inner ear through intricate morphogenetic changes accompanied by the differentiation of specialized cell types to fulfill vestibular and auditory functions (Haddon and Lewis, 1996; Rubel and Fritzsch, 2002). Members of the fibroblast growth factor (Fgf) and Wnt/wingless families secreted from surrounding tissues are critical for OEPD induction and differentiation in all vertebrates examined (Alvarez et al., 2003; Anwar et al., 2017; Freter et al., 2008; Ladher et al., 2005; Léger and Brand, 2002; Maroon et al., 2002; McCarroll et al., 2012; Phillips et al., 2001; Tambalo et al., 2020; Wright and Mansour, 2003). Within the OEPD, members of the forkhead-box (Fox) and distal-less (Dlx) homeodomain transcription factor families are most critical. Foxi1 and the functional homolog Foxi3 provide competence of OEPD formation in zebrafish and amniotes, respectively (Birol et al., 2016; Khatri et al., 2014; Nissen et al., 2003; Solomon et al., 2003). Moreover, OEPD formation depends on Dlx3b and Dlx4b (Dlx3b/4b) in zebrafish and combined loss of *foxi1* and *dlx3b/4b* eliminates all indications of otic specification (Hans et al., 2004; Solomon and Fritz, 2002). At vesicle stages, absence of Dlx3b/4b causes a loss of early-born hair cells (also known as tether cells) and otoliths, large, solidified bio-crystals that mediate vestibular function (Millimaki et al., 2007; Schwarzer et al., 2017). Since the loss of Dlx3 results in early embryonic lethality in mice, the role of Dlx3, which is dynamically expressed in the OEPD in amniotes, has not been addressed so far (Brown et al., 2005; Chen et al., 2017; Morasso et al., 1999). However, depletion of Dlx3 via morpholino-mediated knockdown in chicken shows that *Dlx3* is required for proper otic placode morphogenesis (Uribe et al., 2015). The downstream targets regulated by either Foxi1 and Foxi3 or Dlx3b/4b and Dlx3 are currently completely unknown. Comparative studies using transcriptome data from different organisms will be required to elucidate core vertebrate OEPD gene hierarchies and species-specific pathways. In this context, two otic transcriptome datasets have been generated. One revealed the transcriptome of the developing inner ear from OEPD to placodal stages (Chen et al., 2017). A second identified Fgf-dependent and Fgf-independent pathways occurring during otic placode induction (Yang et al., 2013).

Here, we present a transcriptome dataset of wild-type and Dlx3b/4b-depleted zebrafish embryos using the *pax8*:DsRed transgene, which labels the developing OEPD and kidney anlagen (Ikenaga et al., 2011). At late OEPD stages (6-9-somites or 12-13.5 h post fertilization, hpf), we performed fluorescence-activated cell sorting and the isolated transcripts from sorted cells of both samples were used for transcriptome profiling. Comparison of wild-type versus Dlx3b/4b-depleted samples reveals numerous differentially expressed genes. The most represented gene ontology terms refer to cilium movement, microtubule-based movement, axonemal dynein complex assembly and biomineral tissue development consistent with defects in sensory hair cell and otolith formation displayed in *dlx3b/4b* mutants at otic vesicle stages. A second transcriptome dataset obtained from wild-type and Atoh1b-depleted zebrafish embryos confirms the presence of an Atoh1b-dependent gene set. Using loss-of-function studies, we confirm that Atoh1b controls the proper expression of most of these genes and the subsequent formation of early-born sensory hair cells. Finally, we show that Atoh1b is not only required but also sufficient for the proper onset of gene expression and that the formation of early-born sensory hair cells can be rescued even in the absence of Dlx3b/4b.

## RESULTS

### Transcriptome profiling of wild-type and Dlx3b/4b-depleted embryos reveals numerous differentially regulated genes

**Fig. 1.**
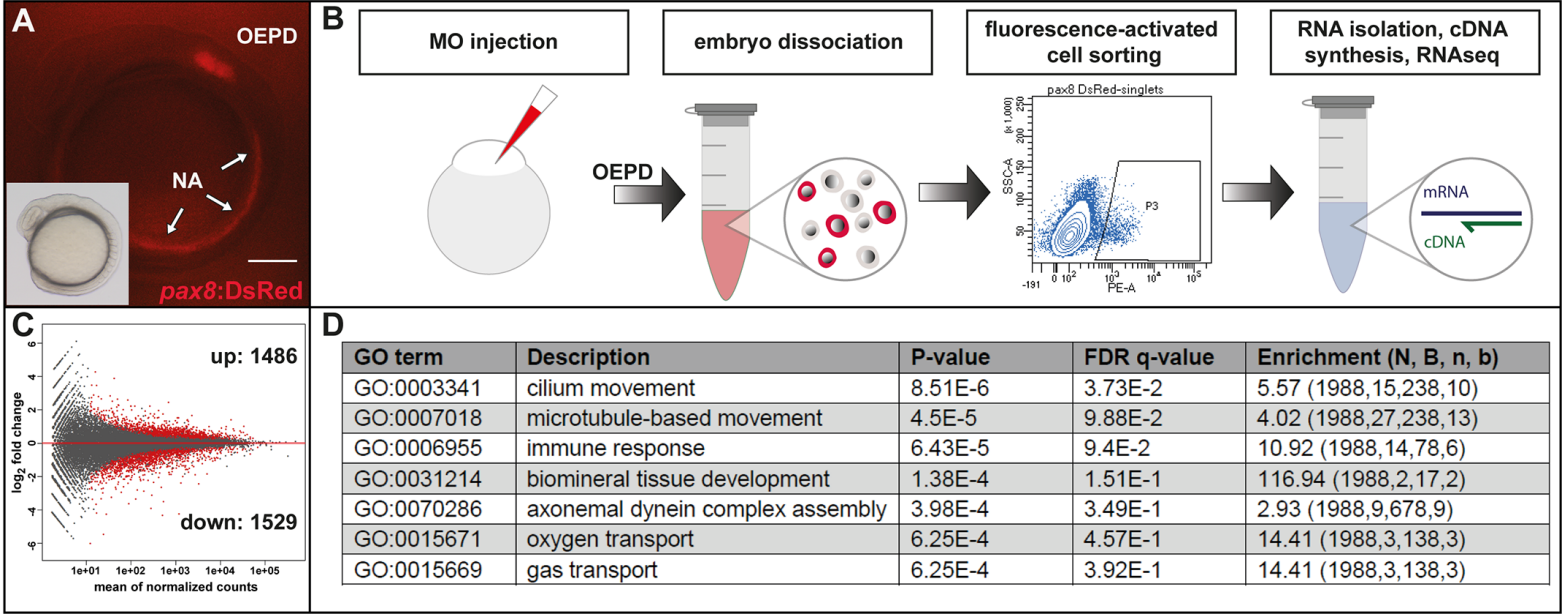
**Transcriptome analysis identifies genes regulated by Dlx3b/4b.** (A) Expression of *pax8*:DsRed in the otic-epibranchial progenitor domain (OEPD) and the nephric anlagen (NA) at the eight-somite stage (13 hpf). Lateral view. Scale bar: 250 µm. (B) Schematic illustration of the experimental procedures. Following *dlx3b/4b* morpholino injection at the one-cell stage, wild-type and *dlx3b/4b* morpholino-injected embryos were dissociated at late OEPD stages (12-13.5 hpf) followed by fluorescence-activated cell sorting. Subsequently, isolated transcripts from sorted, *pax8*:DsRed-positive cells were reverse transcribed into cDNA and used for transcriptome analysis (RNAseq). (C) Comparison of wild-type versus Dlx3b/4b-depleted samples with a false discovery rate of 10% revealed 3015 differentially expressed genes. 1486 and 1529 genes were up- and downregulated respectively. (D) Gene ontology (GO) analysis of the differentially expressed genes using the GOrilla gene ontology analysis tool (Eden et al., 2009). For more information see Material and Methods.

In order to generate a zebrafish otic-enriched gene dataset, we employed heterozygous embryos of the *pax8*:DsRed transgene (Ikenaga et al., 2011), which labels the OEPD and nephric anlagen at early segmentation stages (Fig. 1A). To address the role of Dlx3b/4b specifically, we examined the genes expressed in the presence and absence of Dlx3b/4b. To this aim, we could not use *dlx3b/4b* mutants because *dlx3b/4b* mutants are embryonic lethal and can only be obtained from incrosses of heterozygous animals carrying the deletion allele of *dlx3b/4b* (Schwarzer et al., 2017). However, *dlx3b/4b* mutants do not show any phenotype and are indistinguishable from their wild-type siblings at OEPD stages, meaning we were unable to separate embryos from each other at this time point. Hence, we used depletion of Dlx3b/4b function via injection of antisense morpholino oligomers at the one-cell stage. Importantly, *dlx3b/4b* morpholino injections fully recapitulate the *dlx3b/4b* mutant phenotype (Schwarzer et al., 2017). Moreover, morpholino-mediated knockdown of Dlx3b/4b does not interfere with the expression of *pax8* (Solomon and Fritz, 2002) and the *pax8*:DsRed reporter is properly expressed even in the complete absence of *dlx3b/4b* (Hans et al., 2013). At late OEPD stages (6-9-somites or 12-13.5 hpf), wild-type and *dlx3b/4b* morpholino-injected embryos were dissociated prior to fluorescence-activated cell sorting to gate for live, single cells, out of which DsRed-positive cells were sorted and collected (Fig. 1B and Fig. S1). Subsequently, the isolated transcripts from sorted cells were used for RNA sequencing (RNAseq). Analysis of the read counts of known marker genes in wild-type samples showed that genes expressed during early inner ear and kidney development are highly enriched in the *pax8*:DsRed-sorted cells whereas genes associated with forebrain and muscle development are almost absent (Fig. S2A and Table S1). Similarly, downstream genes of the Fgf signaling pathway are highly overrepresented in *pax8*:DsRed-sorted cells, demonstrating the specificity of the fluorescence-activated cell sorting. Bioinformatic comparison using Euclidean distance and principal component analysis showed that the three biological wild-type and three biological Dlx3b/4b-depleted samples cluster together (Fig. S2B). Applying a false discovery rate of 10%, the comparison of wild-type versus Dlx3b/4b-depleted samples revealed 3.015 differentially expressed genes (DEG) in total, with 1.486 being up- and 1.529 being downregulated (Fig. 1C and Table S2). Gene ontology (GO) analysis using the GOrilla gene ontology analysis tool (Eden et al., 2009) revealed cilium movement, microtubule-based movement, axonemal dynein complex assembly and biomineral tissue development among the most represented GO terms consistent with defective sensory hair cell and otolith formation in *dlx3b/4b* mutants (Fig. 1D and Fig. S2C). The GO analysis using all DEGs revealed mostly downregulated genes associated with the corresponding GO terms. We hence performed a separate GO analysis for upregulated genes only. Interestingly, this analysis revealed mostly GO terms associated with mesodermal tissue development (Fig. S3A).

**Fig. 2.**
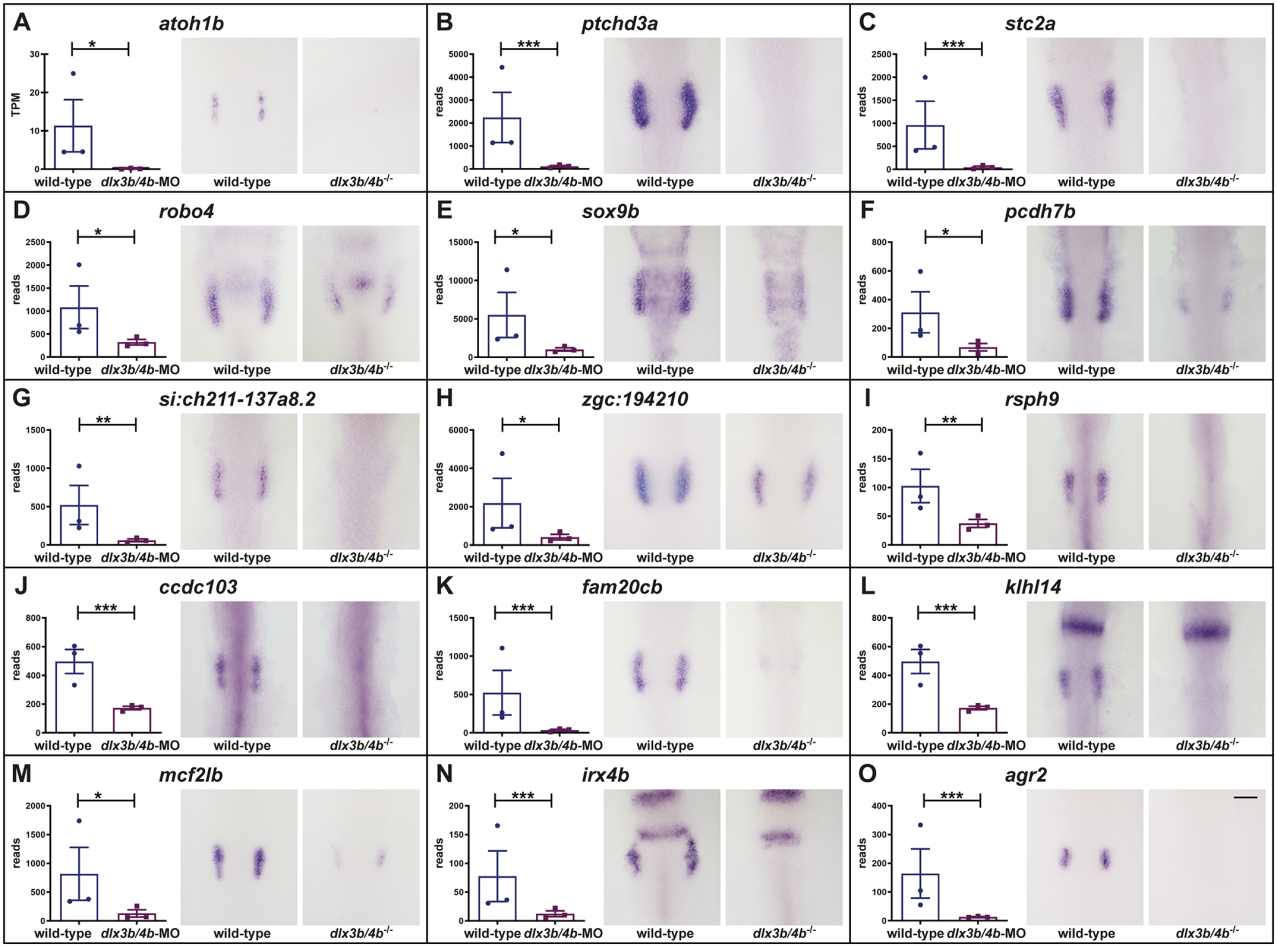
**Dlx3b/4b controls the expression of numerous known and novel transcripts in the OEPD.** In comparison to wild-type siblings, RNAseq and *in situ* hybridization show a downregulation of *atoh1b* (A), *ptchd3a* (B), *stc2a* (C), *robo4* (D), *sox9b* (E), *pcdh7b* (F), *si:ch211-137a8.2* (G), *zgc:194210* (H), *rsph9* (I), *ccdc103* (J), *fam20cb* (K), *klhl14* (L), *mcf2lb* (M), *irx4b* (N) and *agr2* (O) in the OEPD of *dlx3b/4b* mutants at the six-somite stage (12 hpf). Dorsal views, anterior to the top. Scale bar: 100 µm. Except for *atoh1b*, plots show the read counts of the individual genes including standard deviation. Expression levels of *atoh1b* are given in transcripts per million (TPM) because it is annotated to belong to chrUn_KN150642v1 and mapping was done only against known chromosomes. Significance was based on the padj values which were calculated to control the false discovery rate and was assigned to the following ranges: ***: 0-0.001; **: 0.001-0.01; *: 0.01-0.01; not significant: 0.1-1.0.

To validate our RNAseq data, we performed *in situ* hybridization on embryos at late OEPD stages (6-9-somites) obtained from incrosses of heterozygous animals carrying the deletion allele of *dlx3b/4b* (Schwarzer et al., 2017). Analysis of upregulated genes, like *aldh1a2* and *etv5b*, did not corroborate the RNAseq data, presumably due to only moderate upregulation, which is not resolved by *in situ* hybridization and rather masked by the endogenous gene expression (Fig. S3B). Hence, we turned towards downregulated DEGs and analyzed more than 30 downregulated DEGs that either showed a high log2 fold change and a read count >100 or represented known otic genes. So far only *atoh1b* has been shown to be completely lost in the OEPD in the absence of Dlx3b/4b (Millimaki et al., 2007). This finding was confirmed by our RNAseq data and *in situ* hybridization in which a complete loss of *atoh1b* was observed in a quarter of the embryos (Fig. 2A). *In situ* hybridization of known genes like *ptchd3a*, *stc2a*, *robo4*, *sox9b* and *pcdh7b* as well as novel transcripts like *si:ch211-137a8.2* (the human orthologue that has been implicated in autosomal recessive nonsyndromic deafness 76) and *zgc:194210* corroborated our RNAseq data and displayed either a complete loss or severe reduction of the respective gene in a quarter of the embryos (Fig. 2B-H). Transcripts associated with GO terms related to cilium movement and microtubule-based movement, like *rsph9*, axonemal dynein complex assembly, like *ccdc103*, or biomineral tissue development, like *fam20cb*, were also absent in the OEPD of *dlx3b/4b* mutants, as expected from our RNAseq data (Fig. 2I-K). Interestingly, several genes including *klhl14*, *mcf2lb*, *irx4b* and *agr2* which orthologues have been identified to be expressed within the chicken OEPD (Chen et al., 2017), were also confirmed to be regulated by Dlx3b/4b (Fig. 2L-O). To investigate whether additional genes are expressed in the OEPD of both species, we compared our gene set of downregulated DEGs with the otic-enriched transcripts identified in the chick OEPD provided in Table S2 from Chen et al., 2017. To enable a comparison, we used the online tool g:Profiler (Raudvere et al., 2019), in which the orthology search g:Orth translates gene identifiers between organisms and provides orthologous gene mappings based on the information retrieved from Ensembl. The before mentioned Table S2 from Chen et al., 2017 contains in total 2016 entries with Ensembl gene IDs which corresponded to 1846 transcripts with an ensgal gene ID (Fig. S4). Our list of downregulated DEGs comprises 1529 genes in total converting into 1172 genes with a respective ensgal gene ID. The subsequent comparison (1846 versus 1172) revealed 114 genes that are regulated by Dlx3b/4b in zebrafish as well as are expressed in the OEPD of chicken (Table S3). Taken together, we obtained a zebrafish OEPD-specific gene set that comprises genes regulated by the transcription factors Dlx3b/4b. Moreover, 114 genes are also found to be expressed during early avian inner ear development indicating the existence of a presumptive vertebrate gene module active during early inner ear development.

### Dlx3b/4b regulates an Atoh1b-dependent gene set

**Fig. 3.**
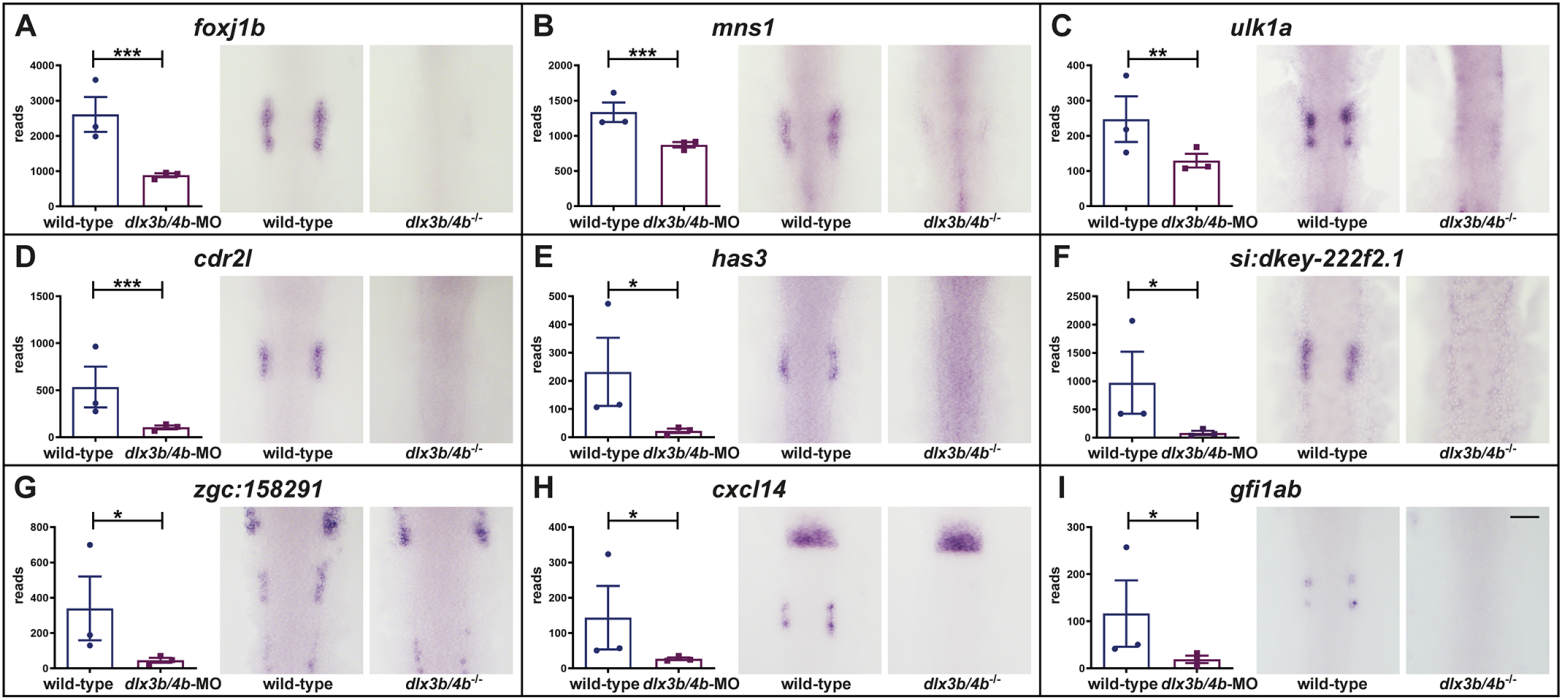
**Dlx3b/4b controls the expression of genes mimicking the expression pattern of *atoh1b*.** Similar to *atoh1b*, *foxj1b* (A), *mns1* (B), *ulk1a* (C), *cdr2l* (D), *has3* (E), *si:dkey-222f2.1* (F), *zgc:158291* (G), *cxcl14* (H) and *gfi1ab* (I) show a restricted expression in the OEPD. In comparison to wild-type siblings, RNAseq and *in situ* hybridization at the six-somite stage (12 hpf) show downregulation of all aforementioned genes in the OEPD of *dlx3b/4b* mutants. Dorsal views, anterior to the top. Scale bar: 100 µm. Plots show the read counts of the individual genes including standard deviation. Significance was based on the padj values which were calculated to control the false discovery rate with the following ranges: ***: 0-0.001; **: 0.001-0.01; *: 0.01-0.01; not significant: 0.1-1.0.

In the course of the validation using *in situ* hybridization, we noticed that the expression of many DEGs is highly reminiscent of the dynamic expression of *atoh1b*. Atoh1b is required for the formation of early-born hair cells (also known as tether cells) that seed and anchor the formation of otoliths, large solidified bio-crystals that mediate vestibular function (Millimaki et al., 2007; Schwarzer et al., 2017). In contrast to *pax8*, *pax2a* and *dlx3b* which are expressed in the entire OEPD, *atoh1b* displays a restricted expression. It is initially expressed in a single domain abutting the hindbrain and becomes progressively constricted to two separate patches that correspond to the future anterior and posterior prosensory domains (Millimaki et al., 2007; Radosevic et al., 2014). In addition to *foxj1b*, a known downstream target of Atoh1b within the OEPD (Yu et al., 2011), *in situ* hybridization of further genes like *mns1*, *ulk1a*, *cdr2 l*, *has3*, *cxcl14* and *gfi1ab* as well as novel transcripts like *si:dkey222f2.1* and *zgc:158291* showed an *atoh1b*-like OEPD expression and were completely absent in the OEPD of *dlx3b/4b* mutant embryos (Fig. 3A-I).

To further investigate whether these DEGs are not only expressed similarly to *atoh1b* but are actually regulated by Atoh1b, we repeated the experiment shown in Fig. 1B but this time in the presence and absence of Atoh1b. Depletion of Atoh1b function was achieved via morpholino injection at the one-cell stage (Millimaki et al., 2007). Again, wild-type and *atoh1b* morpholino-injected embryos were dissociated at late OEPD stages (6-9-somites) followed by fluorescence-activated cell sorting using the established gating strategy. Subsequently, the isolated transcripts from sorted, *pax8*:DsRed-positive cells were used for RNAseq. Bioinformatic comparison using Euclidean distance and principal component analysis showed that the three biological Atoh1b-depleted samples cluster together (Fig. S5A). In contrast, one wild-type sample diverged significantly from the other two wild-type samples and was therefore dismissed in the further analysis (Fig. S5B). Applying a false discovery rate of 10%, the comparison of wild-type versus Atoh1b-depleted samples revealed 719 DEGs in total, with 438 being upregulated and 281 being downregulated (Fig. S5C and Table S4).

To validate the obtained RNAseq data with *in situ* hybridization, we established an unambiguous null allele of *atoh1b*. To this aim, two CRISPR/Cas9 target sites separated by 1.924 bp up- and downstream of *atoh1b* were chosen to eliminate the entire open reading frame (Fig. S6A). Following establishment of the deletion, *in situ* hybridization against *atoh1b* in embryos at 24 hpf obtained from incrosses of heterozygous carriers showed a complete loss of *atoh1b* mRNA in a quarter of the clutch, corroborating the absence of the *atoh1b* gene (Fig. S6B). Consistent with the previous phenotypic description following *atoh1b* morpholino injection (Millimaki et al., 2007), a single, initially untethered otolith was observed in a quarter of the embryos indicating that the *atoh1b* morpholino reliably phenocopies the *atoh1b* loss-of-function mutation with respect to inner ear development (Fig. S6C). The single otolith becomes tethered after 30 hpf and genotyping confirms that the single otolith phenotype harbors the *atoh1b* deletion allele only (Fig. S6D). In contrast, randomly selected embryos with wild-type morphology contained the wild-type allele either in homozygosity or in combination with the *atoh1b* deletion allele. The newly established *atoh1b* deletion allele was subsequently used to validate the RNAseq data.

**Fig. 4.**
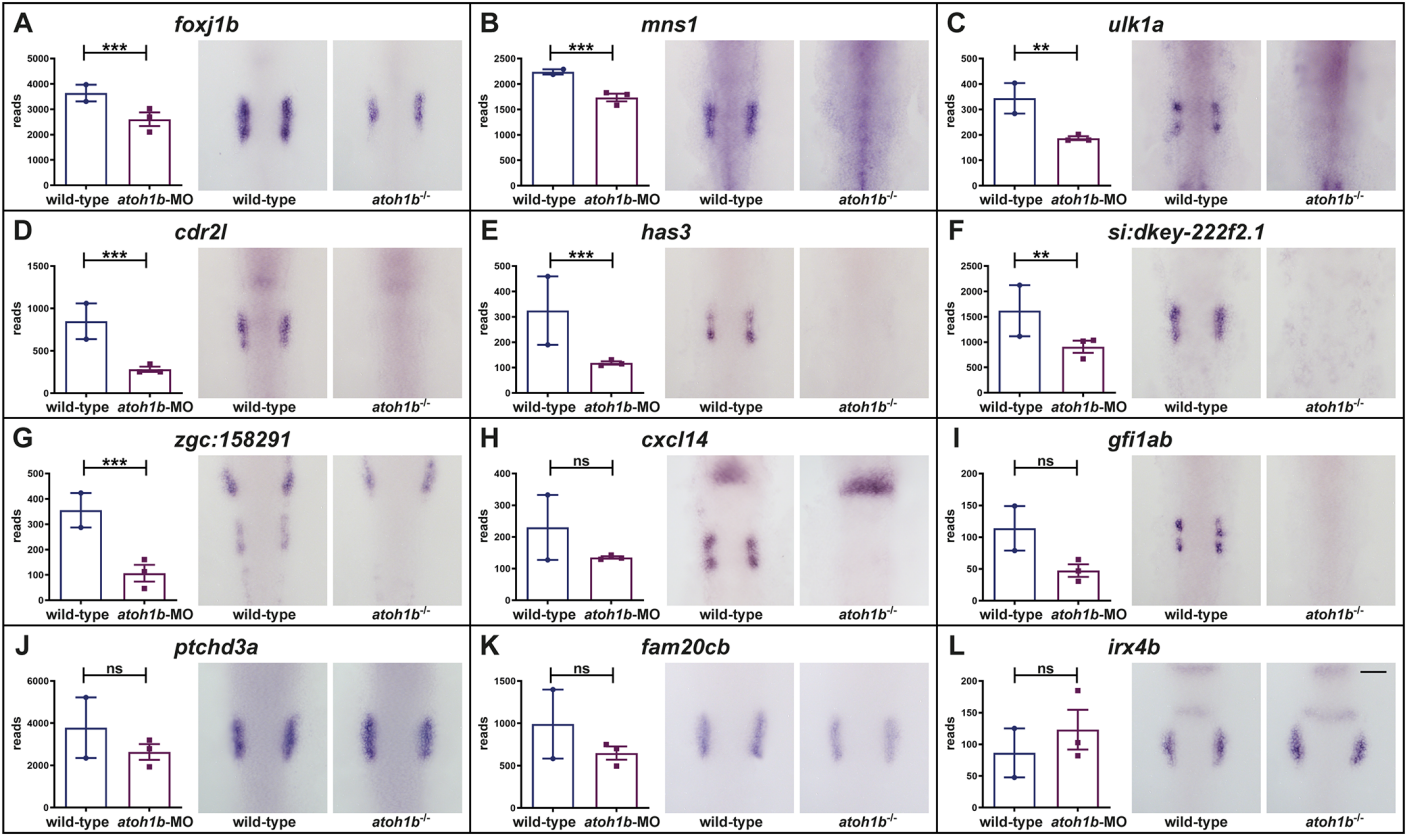
**Atoh1b controls the expression of numerous genes in the OEPD.** In comparison to wild-type siblings, RNAseq and *in situ* hybridization show downregulation of *foxj1b* (A), *mns1* (B), *ulk1a* (C), *cdr2l* (D), *has3* (E), *si:dkey-222f2.1* (F) and *zgc:158291* (G) in the OEPD of *atoh1b* mutants. Expression of *cxcl14* (H) and *gfi1ab* (I) is also absent in the OEPD of *atoh1b* mutants although RNAseq indicates a trend but no significant difference. Expression of *ptchd3a* (J), *fam20cb* (K) and *irx4b* (L) are not affected in *atoh1b* mutants neither based on the RNAseq data nor by *in situ* hybridization. Dorsal views, anterior to the top. Scale bar: 100 µm. Plots show the read counts of the individual genes including standard deviation. Significance was based on the padj values which were calculated to control the false discovery rate with the following ranges: ***: 0-0.001; **: 0.001-0.01; *: 0.01-0.01; not significant: 0.1-1.0.

To this aim, *in situ* hybridization was performed on embryos at late OEPD stages (6-9-somites) obtained from incrosses of heterozygous *atoh1b* carriers. Consistent with the RNAseq data, *foxj1b*, *mns1*, *ulk1a*, *cdr2l*, *has3*, *si:dkey222f2.1* and *zgc:158291* were severely reduced or completely absent in the OEPD of *atoh1b* mutant embryos (Fig. 4A-G). Moreover, also *cxcl14* and *gfi1ab* transcripts could not be detected in the OEPD of *atoh1b* mutant embryos, although RNAseq indicated reduced expression but did not identify them as significantly regulated genes (Fig. 4H, I). In contrast, other Dlx3b/4b-regulated genes with widespread OEPD expression like *ptchd3a*, *fam20cb* or *irx4b* did not show any differential gene expression neither based on RNAseq nor with *in situ* hybridization (Fig. 4J-L). Finally, to identify additional genes downstream of Dlx3b/4b and Atoh1b, we compared the downregulated DEGs following Dlx3b/4b depletion with the downregulated DEGs following Atoh1b depletion. Whereas the former list contained 1529 DEGs, the latter contained only 281 DEGs. The subsequent comparison revealed 52 genes to be downregulated following Dlx3b/4b as well as Atoh1b depletion (Fig. S7 and Table S5). Taken together, our results show the existence of an Atoh1b-regulated gene set.

### Ectopic Atoh1b expression rescues sensorigenesis in the absence of Dlx3b/4b

**Fig. 5.**
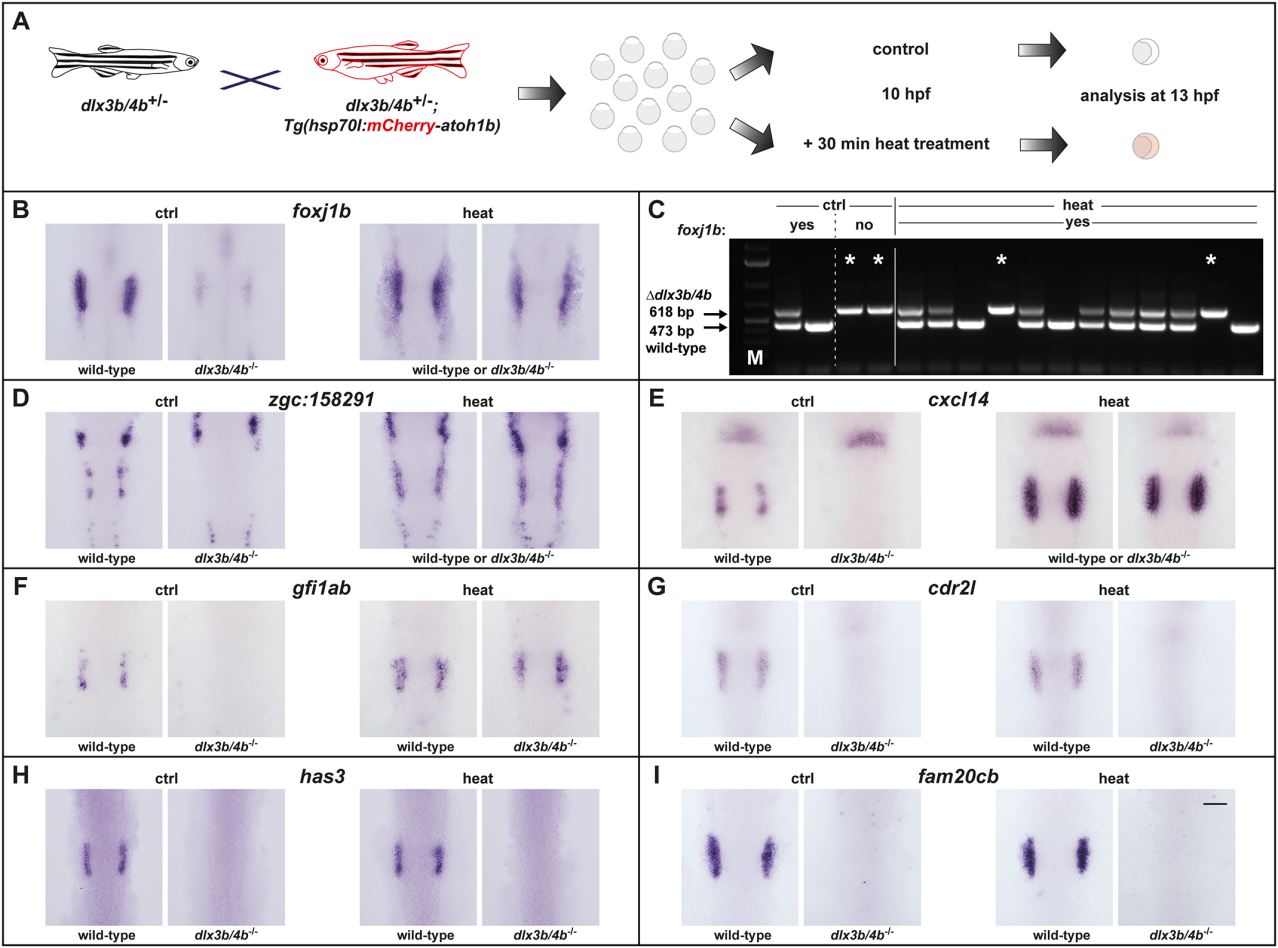
**Ectopic Atoh1b rescues gene expression in the OEPD in the absence of Dlx3b/4b.** (A) Scheme of the experimental outline. Progeny were obtained from heterozygous animals carrying the *dlx3b/4b* deletion allele (*dlx3b/4b*^+/−^) crossed with animals carrying the *dlx3b/4b* deletion allele as well as the transgene to misexpress *atoh1b* conditionally [*dlx3b/4b*^+/−^;*Tg(hsp70l:mCherry-T2a-atoh1b)*], both in heterozygosity. At 10 hpf, the clutch was split and either treated with heat or served as untreated control. At 13 hpf, corresponding to late OEPD stages, heat-treated embryos were sorted for mCherry fluorescence and subsequently fixed for analysis just as untreated controls. (B) In untreated controls (ctrl), *foxj1b* is robustly expressed in the majority of the embryos (wild-type) whereas one quarter displays a significant downregulation (*dlx3b/4b*^−/−^). In contrast, all heat-treated embryos (heat), show a widespread *foxj1b* expression, even extending the common OEPD territory. (C) Multiplex PCR reveals the genotype of individual embryos following *in situ* hybridization. *dlx3b/4b* mutants are indicated with an asterisk. Two embryos of untreated controls with a strong *foxj1b* expression carry the wild-type allele detected with a 473 base pairs (bp) amplicon in homozygosity or in combination with the *dlx3b/4b* deletion allele detected with a 618 bp amplicon. In contrast, two embryos of untreated controls with strongly reduced *foxj1b* expression show only presence of the *dlx3b/4b* deletion allele (asterisk). Out of 12 heat-treated embryos with strong *foxj1b* expression, two embryos carry the *dlx3b/4b* deletion allele only (asterisk). M indicates marker for molecular size standard. (D-F) Expression of *zgc:158291* (D), *cxcl14* (E) and *gfi1ab* (F) behave similar to *foxj1b* in response to misexpression of *atoh1b*. (G,H) Expression of *cdr2l* (G) and *has3* (H) is unchanged after misexpression of *atoh1b*. (I) Similarly, ectopic expression of *atoh1b* is not able to rescue *fam20cb* expression. (B, D-I) Dorsal views, anterior to the top. Scale bar: 100 µm.

To further analyze the Atoh1b-regulated genes, we generated a stable transgenic line that allowed us to express *atoh1b* conditionally throughout the entire embryo. To this aim, a single open reading frame coding for mCherry and Atoh1b separated by the viral T2A peptide sequence was placed under the control of the zebrafish temperature-inducible *heat shock cognate 70-kd protein, like* (*hsp70l*) promoter (Fig. S8A). We tested the reliability of the line by monitoring mCherry and *atoh1b* expression in transgenic animals before and after heat shock. At permissive temperatures, no expression of fluorescent mCherry is observed. In contrast, strong and ubiquitous mCherry is present following 2-3 h post heat treatment at 28 hpf (Fig. S8B). To monitor the activity of the *hsp70l* promoter after completion of the heat treatment in more detail, we performed heat treatments at the end of gastrulation (10 hpf) just prior to the onset of endogenous *atoh1b* expression in the OEPD. Prior to heat treatment, all embryos from a cross between heterozygous *hsp70l:mCherry-T2a-atoh1b* and wild-type fish show no *atoh1b* expression (Fig. S8C). Following a 30 min heat treatment, we observe strong and ubiquitous expression of *atoh1b* in approximately 50% of the progeny. Expression levels of *atoh1b* under these conditions are very high and mask endogenous *atoh1b* expression. Ectopic *atoh1b* transcripts are gradually lost within approximately 4.5 h and heat-treated transgenic embryos are indiscernible from non-transgenic siblings with respect to *atoh1b* expression at 5 h post heat treatment (Fig. S8C). However, persistent mCherry fluorescence still enables easy identification of heat-treated, transgenic embryos. Hence, heat treatment results in a transient but strong and ubiquitous overexpression of *atoh1b* in mCherry-labelled embryos. To address the function of the Atoh1b-regulated genes, we combined the *hsp70l:mCherry-T2a-atoh1b* transgene with the *dlx3b/4b* deletion allele and performed crosses with heterozygous animals carrying the *dlx3b/4b* deletion allele only (Fig. 5A). The obtained progeny were raised to 10 hpf and split into two groups. One group underwent a 30 min heat treatment and the second group served as the untreated control. Prior to fixation of both samples at 3 h post heat treatment, mCherry-negative embryos were removed from the heat-treated sample. Subsequently, *in situ* hybridization with the identified Atoh1b-dependent genes was performed with both samples. As expected by the Mendelian laws of inheritance, expression of *foxj1b* was downregulated within the OEPD in one quarter of the embryos in the untreated control sample (Fig. 5B, Table 1). In contrast, all embryos of the heat-treated sample displayed not only *foxj1b* expression within the OEPD but also ectopically in the preplacodal region anterior and posterior to the OEPD. The fact that all embryos showed *foxj1b* expression indicated that ectopic Atoh1b activity is able to rescue *foxj1b* expression even in the absence of Dlx3b/4b. To corroborate this finding, we genotyped embryos following *in situ* hybridization using our previously established multiplex PCR (Fig. S9) (Schwarzer et al., 2017). Genotyping of two embryos from the untreated sample with regular *foxj1b* expression displayed the wild-type allele shown by the presence of a 473 base pair (bp) fragment either in homozygosity or in combination with the *dlx3b/4b* deletion allele shown by a 618 bp amplicon (Fig. 5C). Two embryos from the same untreated sample but lacking *foxj1b* expression displayed the presence of the *dlx3b/4b* deletion allele only. Genotyping of twelve randomly selected embryos with strong *foxj1b* expression from the heat-treated sample identified them as wild-type and heterozygous. However, also two embryos harboring the *dlx3b/4b* deletion allele only were identified. Further analysis of *zgc:158291*, *cxcl14* and *gfi1ab* resulted in the same finding, although strong expression of *cxcl14* and *gfi1ab* was restricted to the OEPD and only *zgc:158291* showed an ectopic expression in the preplacodal region anterior and posterior to the OEPD similar to *foxj1b* (Fig. 5D-F, Table 1). Interestingly, not all Atoh1b-regulated genes were rescued and the ratio of embryos with and without *has3* and *cdr2l* expression was similar in untreated control and heat-treated samples (Fig. 5G,H, Table 1). As expected, ectopic expression of *atoh1b* did not rescue the expression of *fam20cb* which displays an OEPD-wide expression and is associated with the GO term biomineral tissue development (Fig. 5I, Table 1).

**Table 1.**
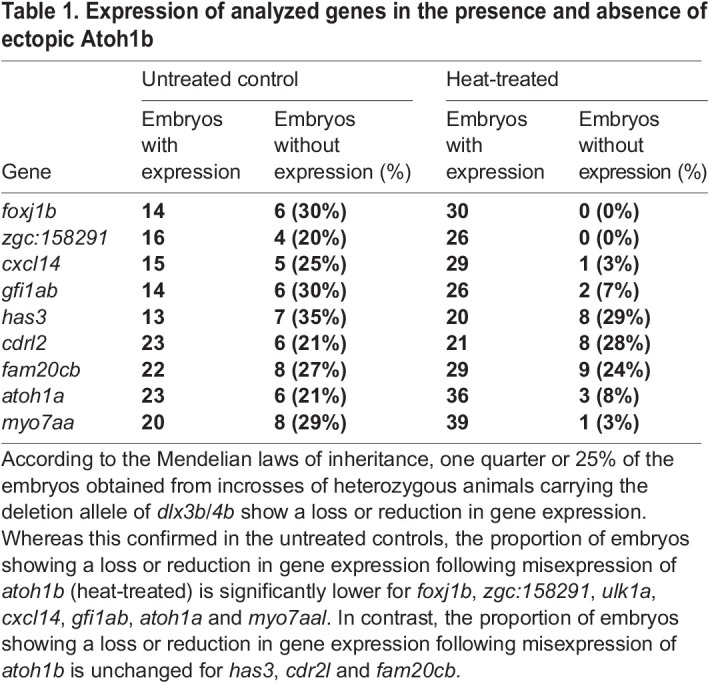
Expression of analyzed genes in the presence and absence of ectopic Atoh1b

**Fig. 6.**
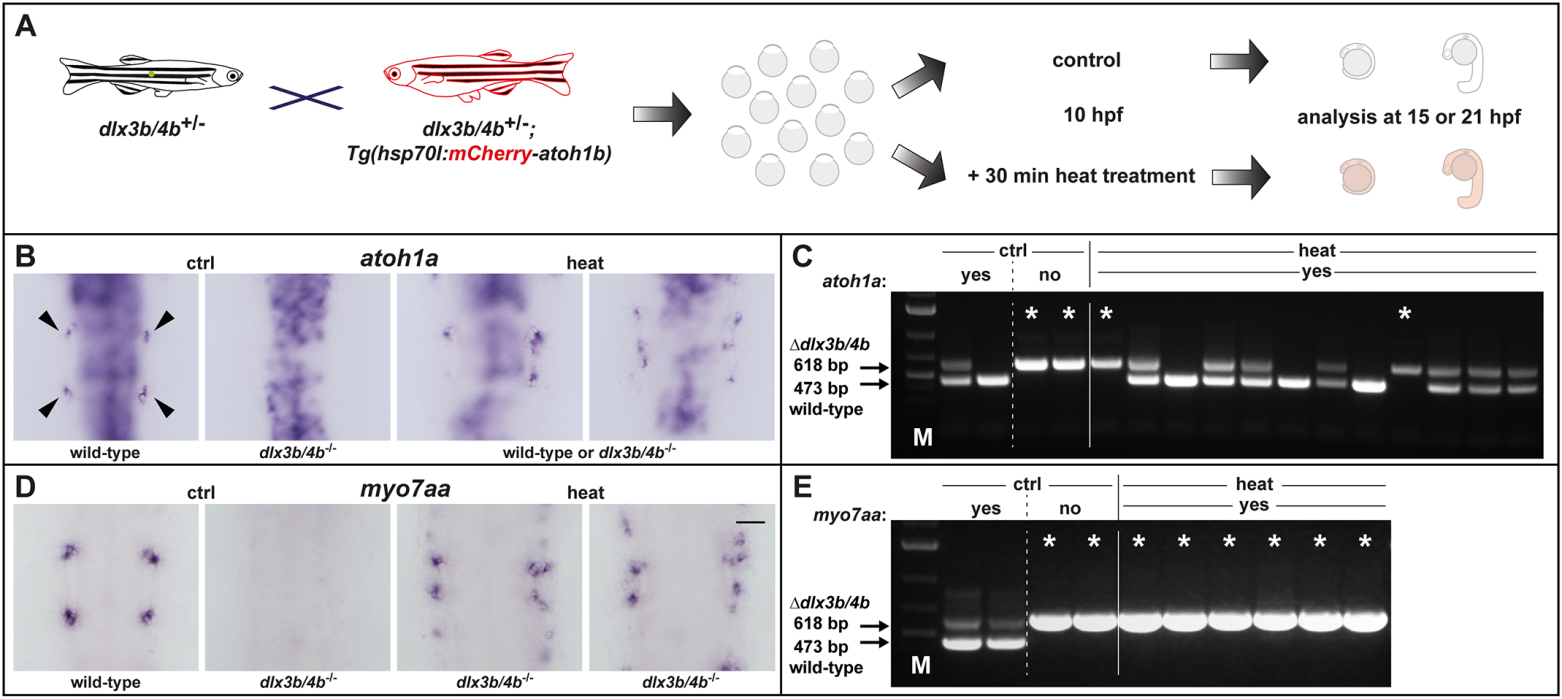
**Ectopic Atoh1b rescues otic sensorigenesis in the absence of Dlx3b/4b.** (A) Scheme of the experimental outline. Progeny were obtained from heterozygous animals carrying the *dlx3b/4b* deletion allele (*dlx3b/4b*^+/−^) crossed with animals carrying the *dlx3b/4b* deletion allele as well as the transgene to misexpress *atoh1b* conditionally [*dlx3b/4b*^+/−^;*Tg(hsp70l:mCherry-T2a-atoh1b)*], both in heterozygosity. At 10 hpf, the clutch was split and either treated with heat or served as untreated control. At 15 or 21 hpf, corresponding to placodal or vesicle stages, heat-treated embryos were sorted for mCherry fluorescence and subsequently fixed for analysis just as untreated controls. (B) In untreated controls (ctrl) at 15 hpf, *atoh1a* is expressed in the majority of the embryos in discrete anterior and posterior domains of the otic placode (arrows in wild-type) whereas one quarter display a complete absence of *atoh1a* (*dlx3b/4b*^−/−^). In heat-treated embryos (heat), otic *atoh1a* expression is less regular but present in the majority of embryos. (C) Multiplex PCR reveals the genotype of individual embryos following *in situ* hybridization. *dlx3b/4b* mutants are indicated with an asterisk. Two embryos of untreated controls with proper *atoh1b* expression carry the wild-type allele (473 bp amplicon) in homozygosity or in combination with the *dlx3b/4b* deletion allele (618 bp amplicon). Two embryos of the same, untreated controls with no *atoh1b* expression show only presence of the *dlx3b/4b* deletion allele (asterisk). Out of 12 heat-treated embryos with *atoh1b* expression, two embryos carried the *dlx3b/4b* deletion allele only (asterisk). M indicates marker for molecular size standard. (D) Expression of the hair-cell marker *myo7aa* is present anteriorly and posteriorly in the otic vesicle of untreated, wild-type controls at 21 hpf. In contrast, *myo7aa* expression is absent in *dlx3b/4b* mutant embryos, which can be recognized based on smaller otic vesicles. In the heat-treated sample, *myo7aa* expression is found in several embryos with reduced otic vesicles. (E) Multiplex PCR corroborates the finding following *in situ* hybridization. *dlx3b/4b* mutant embryos are indicated with an asterisk. Two embryos of untreated controls with proper *myo7aa* expression carry the wild-type allele in combination with the *dlx3b/4b* deletion allele. Two embryos of the same, untreated control sample with no *myo7aa* expression show only presence of the *dlx3b/4b* deletion allele (asterisk). All six embryos with a reduced otic vesicle size but detectable *myo7aa* expression carry the *dlx3b/4b* deletion allele only (asterisk). M indicates marker for molecular size standard. (B,D) Dorsal views, anterior to the top. Scale bar: 75 µm.

One of the analyzed genes, the transcription factor Gfi1, has been shown to be required for hair cell differentiation and survival (Wallis et al., 2003). Hence, we looked into subsequent sensorigenesis in more detail. To this aim, we repeated the previous experiment using the *hsp70l:mCherry-T2a-atoh1b* transgene in the background of the *dlx3b/4b* deletion allele. Again, the obtained progeny were raised to 10 hpf and split into two groups. One group underwent a 30 min heat treatment and the second group served as the untreated control. mCherry-negative embryos were removed from the heat-treated sample prior to fixation of the samples at 15 and 21 hpf, which corresponds to placodal and vesicle stages, respectively (Fig. 6A). We first analyzed expression of *atoh1a*, which is initiated in discrete anterior and posterior domains of the otic placode at 14 hpf corresponding to the prospective utricular and saccular maculae of the otic vesicle (Millimaki et al., 2007). As expected, we found a loss of *atoh1a* expression within the otic lineage in one quarter of the embryos in the untreated control sample (Fig. 6B, Table 1). In contrast, the vast majority of embryos of the heat-treated sample displayed *atoh1a* expression within the otic territory. This indicated that ectopic Atoh1b activity is also able to rescue *atoh1a* expression even in the absence of Dlx3b/4b. To confirm this finding, we genotyped embryos from both samples following *in situ* hybridization. Similar to *foxj1b*, we found that presence and absence of otic *atoh1a* expression in embryos of the untreated control sample depends on the presence and absence of the *dlx3b/4b* wild-type allele (473 bp fragment), respectively (Fig. 6C). Genotyping of twelve randomly selected embryos with otic *atoh1b* expression from the heat-treated sample, however, revealed the presence of two embryos showing otic *atoh1a* expression despite harboring the *dlx3b/4b* deletion allele only (618 bp fragment). To investigate if even early-born hair cells (also known as tether cells) are also formed in the absence of Dlx3b/4b, we analyzed *myosin VIIAa* (*myo7aa*) expression at 21 hpf (Ernest et al., 2000). At this stage, *myo7aa* is expressed in discrete anterior and posterior cells of the otic vesicle corresponding to the prospective utricular and saccular maculae in wild-type embryos. In contrast, *myo7aa* expression is absent in otic vesicles of *dlx3b/4b* mutants which can be easily identified based on their significantly smaller otic vesicles due to compromised otic induction (Schwarzer et al., 2017). Consistently, we found in the untreated control sample that all embryos with a wild-type-sized otic vesicle showed proper *myo7aa* expression, whereas one quarter of embryos displaying smaller otic vesicles lacked *myo7aa* expression (Fig. 6D, Table 1). In the heat-treated sample however, almost all embryos displayed otic *myo7aa* expression, even embryos with a reduced otic vesicle size. This finding was corroborated using genotyping. Again, otic expression of *myo7aa* in untreated specimen was directly linked to the presence of the *dlx3b/4b* wild-type allele (473 bp fragment), whereas presence of the *dlx3b/4b* deletion allele (618 bp fragment) was associated with absence of *myo7aa* in untreated embryos (Fig. 6E). In contrast, genotyping of six embryos displaying smaller otic vesicles but detectable otic *myo7aa* expression from the heat-treated sample revealed that all six embryos were *dlx3b/4b* mutants. Taken together, these data show that Atoh1b expression controls the expression of several early sensory specification genes and that ectopic Atoh1b activity is able to rescue the formation of early-born hair cells even in the absence of Dlx3b/4b.

## DISCUSSION

Formation of the otic placode, a small epithelial thickening adjacent to the developing hindbrain, is the first morphological manifestation of inner ear development. So far, signaling molecules from different families secreted from surrounding tissues have been identified and govern otic fate induction and differentiation. In particular, Fgf signaling is of key importance to initiate the induction process followed by Wnt signaling as otic fate acquisition progresses (Sai and Ladher, 2015). However, the interplay of factors within the OEPD and their hierarchical organization are only beginning to be understood. Genome-wide transcriptome analysis can be used to identify regulatory modules and comparative approaches using different model organisms will shed light to distinguish species-specific as well as core vertebrate modules. In a first approach, a microarray comparison of otic genes versus non-otic ectodermal genes in the chick embryo revealed that FGF signaling is sufficient to activate an initially small number of otic genes (Yang et al., 2013). Subsequent work, supported the Fgf signaling-dependent induction of only a small set of transcription factors that establish positive feedback loops and thereby stabilize otic progenitor identity (Anwar et al., 2017). The most comprehensive transcriptional analysis was provided with the examination of the transcriptome of the chicken inner ear (Chen et al., 2017). In this study, the comparison of the otic transcriptome at preplacodal to placodal stages revealed a hierarchical organized gene regulatory network providing otic identity during development (Chen et al., 2017).

**Fig. 7.**
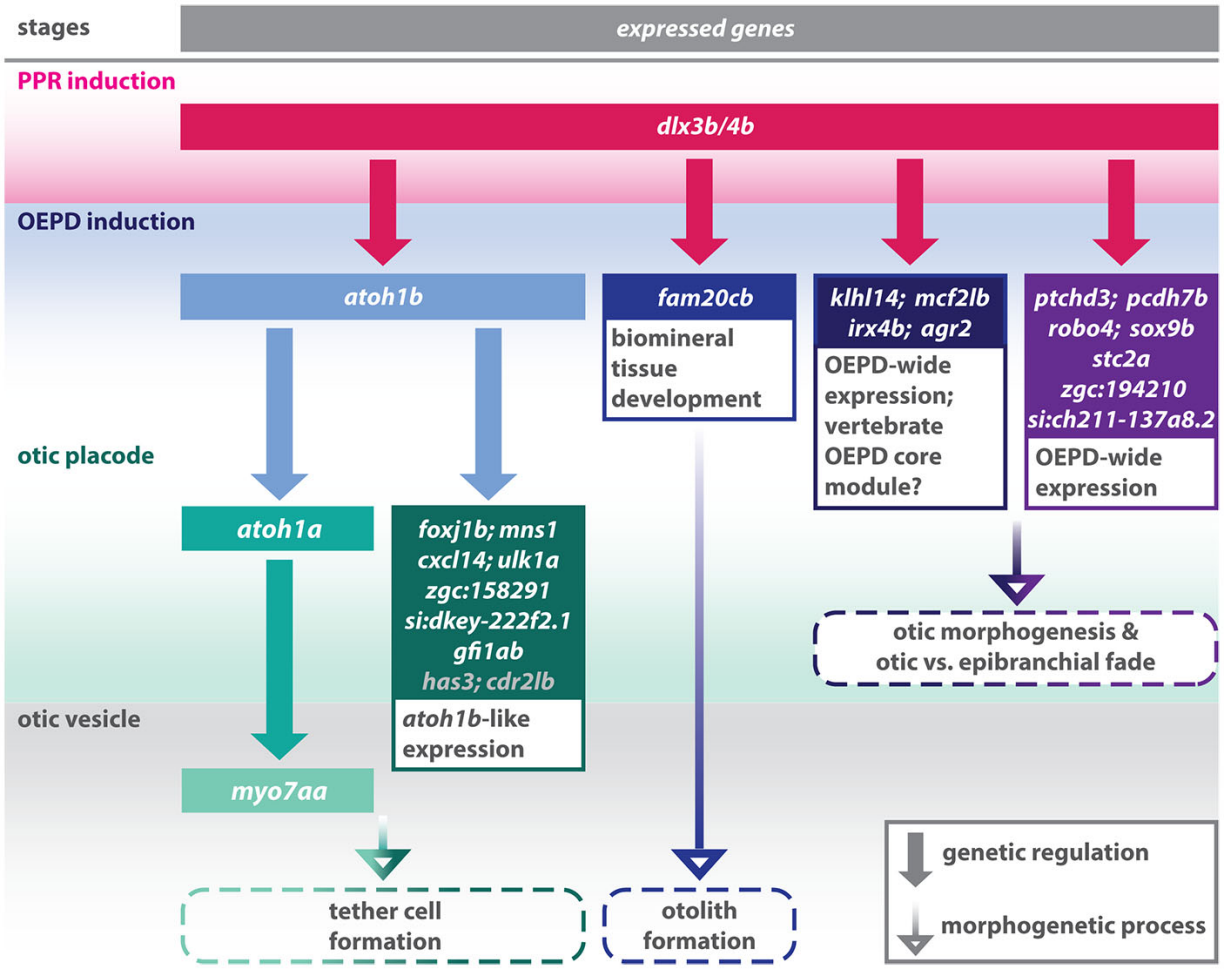
**Schematic summary of the genetic events controlled by Dlx3b/4b during early otic development in zebrafish.** Dlx3b/4b are expressed already during formation of the preplacodal region (PPR). After induction of the otic-epibranchial progenitor domain (OEPD), Dlx3b/4b control the onset of various genes which might govern various morphogenetic processes at subsequent placodal and vesicles stages. Among the genes showing an OEPD-wide expression (*ptchd3*, *pcdh7b*, *si:ch211-137a8.2*, *robo4*, *sox9b*, *stc2a* and *zgc:194210*), we also find *klhl14*; *mcf2lb*; *irx4b* and *agr2* which have been reported to be expressed within the chicken OEPD (Chen et al., 2017). Hence, they might be members of a conserved, vertebrate core module regulating subsequent otic morphogenesis as well as otic versus epibranchial fate decisions. In addition, we find *foxj1b*, *mns1*, *ulk1a*, *cxcl14*, *zgc:158291*, *si:dkey-222f2.1*, *gfi1ab*, *has3* and *cdr2l*, which display an *atoh1b*-like expression and might play a role in tether cell formation. Except *has3* and *cdr2l* (grey), all genes of this module can be activated via ectopic expression of Atoh1b, even in the absence of Dlx3b/4b. Finally, we find that Dlx3b/4b controls the expression of *fam20cb* a gene associated with biomineral tissue development which might be important for subsequent otolith formation.

Here, we provide a zebrafish-derived dataset of genes expressed in the OEPD. Using heterozygous progeny from the *pax8*:DsRed transgenic line (Ikenaga et al., 2011), we were able to generate an otic-enriched gene dataset and address specifically the role of the known competence factors Dlx3b/4b during early inner ear formation. Interestingly, the GO term analysis of upregulated DEGs only revealed mostly GO terms associated with mesodermal tissue development. This indicates that Dlx3b/4b might be involved in the acquisition of proper mesodermal fates at early segmentation stages representing a potentially new but so far unknown function of Dlx3b/4b. Analysis of several selected upregulated DEGs via *in situ* hybridization did not corroborate the RNAseq data. However, this is presumably due to only moderate upregulation, which is not resolved by *in situ* hybridization and is masked by the endogenous gene expression. In contrast, using downregulated DEGs, we identify known and novel transcripts displaying widespread OEPD expression regulated in a Dlx3b/4b-dependent manner (Fig. 7). Gene ontology analysis did not indicate any further functional role of these genes, but they might be required for proper otic morphogenesis and/or subsequent acquisition of otic and epibranchial fates. In this context, a subset of genes is of particular interest as the orthologues show expression in the OEPD of zebrafish and chicken. These genes might hence belong to a conserved, vertebrate OEPD core module. However, further comparative studies will be required to identify all members of this potential module, which we here propose based on only two datasets derived from chick and zebrafish and which might be incomplete. Our analysis also revealed an Atoh1b-controlled gene set downstream of Dlx3b/4b. This gene set was initially identified because the respective genes show a restricted OEPD expression pattern, highly reminiscent to *atoh1b* expression. Subsequent *atoh1b* loss-of-function analysis corroborated the Atoh1b-dependent gene expression. Moreover, the reverse *atoh1b* gain-of-function experiments showed that expression of many genes can be rescued even in the absence of Dlx3b/4b. In this context, it was striking that the rescued gene expression was restricted in most cases to the OEPD. In some cases, ectopic gene activation extended into the preplacodal region located anteriorly and posteriorly to the OEPD but we never observed widespread, ectopic expression in other tissues. Moreover, *atoh1b* misexpression in the absence of Dlx3b/4b was not sufficient to rescue OEPD expression of all Atoh1b-dependent genes. This indicates that either other Dlx3b/4b-dependent transcriptional activators, epigenetic regulators or a combination of both control the expression of OEPD genes at a larger scale. The role of epigenetic factors during early inner development is only beginning to be understood. In this context, loss-of-function studies of lysine-specific demethylase 1a, Kdm1a (also known as Lsd1) and lysine-specific demethylase 4B, Kdm4b, revealed that modulation of histone methylation interfered with proper inner ear morphogenesis (Ahmed and Streit, 2018; Uribe et al., 2015). Moreover, the *Dlx3* locus was shown to be a direct target of Kdm4b (Uribe et al., 2015).

With respect to transcriptional activators, forkhead box transcription factors have been termed pioneer factors because they are able to bind to their target sites and open up the chromatin structure, allowing other transcription factors to bind and activate their targets (Golson and Kaestner, 2016). Consistently, loss of Foxi1, the second competence factor at the top of the otic induction hierarchy in zebrafish, results in delayed onset of *pax2a*, *sox9a*, *sox9b* or *atoh1b* expression in the OEPD (Hans et al., 2013, 2004; Nissen et al., 2003; Solomon et al., 2003). Moreover, and consistent with a function as a pioneer factor, Foxi1 has been shown to remodel the chromatin structure and to remain bound to condensed chromosomes even during mitosis (Yan et al., 2006). Given the importance of Foxi1 and its functional homolog Foxi3 in early otic development in zebrafish and amniotes, respectively, it will be key to determining their respective downstream targets. Unfortunately, our experimental pipeline using morpholino-mediated gene knockdown in heterozygous *pax8*:DsRed transgenic embryos and subsequent transcriptome analysis cannot be applied because onset of *pax8* expression in the OEPD is not only delayed but completely lost in *foxi1* mutants (Nissen et al., 2003; Solomon et al., 2003). Hence, a fluorescent reporter driven by a different promoter will be required. The promoters or promoter fragments of *foxi1* or *dlx3b* represent good candidates because their activity is unaffected by the loss of *foxi1*. Alternatively, use of regulatory elements of OEPD-wide expressed genes downstream of Dlx3b/4b identified in this study (e.g. *ptchd3a*) could also be used.

Our Atoh1b gain-of-function experiments did not only show a rescue of individual gene expressions in the absence of Dlx3b/4b at OEPD stages but also the rescue of sensorigenesis at subsequent stages. Interestingly, we never observed an expansion of sensory fate within the otic lineage following *atoh1b* misexpression which is in line with previous reports (Sweet et al., 2011). Here, the authors showed that misexpression of *atoh1a* at placodal stages is sufficient to induce ectopic sensory hair cells. However, the competence to respond to Atoh1 is temporally and spatially controlled and misexpression of *atoh1a* at earlier stages had no such effect and only showed proper sensorigenesis. Interestingly, although early-born hair cells could be rescued in *dlx3b/4b* mutants following ectopic expression of *atoh1b*, the formation of otoliths still failed. This finding shows that the generation of early-born hair cells and otoliths are both regulated by Dlx3b/4b but are genetically separated subsequently. In zebrafish, seeding of otoliths starts in the lumen of the otic vesicle at 18 hpf with an organic core that acts as a site for nucleation and subsequent biomineralization (Lundberg et al., 2015; Thomas et al., 2019). After seeding, the nascent otoliths attach to the tips of the kinocilia of the early-born hair cells (also known as tether cells) and rapidly grow through deposition of calcium carbonate during development (Riley et al., 1997; Stooke-Vaughan et al., 2012; Tanimoto et al., 2011). In this context, otolith tethering is not a prerequisite for otolith growth as loss-of-function analysis of *otogelin* (*otog*) or *atoh1b* reveal a single, untethered otolith which only becomes tethered at around 28-30 hpf (Millimaki et al., 2007; Stooke-Vaughan et al., 2012, 2015). How otolith nucleation is initiated in detail remains elusive. Based on proteomic data from the inner ear of the black bream (*Acanthopagrus butcheri*), it has been suggested that a putative homologue of Starmaker, an intrinsically disordered protein, and the extracellular serine/threonine protein kinase FAM20C play key roles during the crucial early period of nucleation (Thomas et al., 2019). Consistently, we find that *fam20cb* which is associated with the gene ontology biomineral tissue development is completely lost in *dlx3b/4b* mutant embryos but unaffected in *atoh1b* mutants. Absence of *fam20cb* can also not be rescued via ectopic expression of *atoh1b* and might hence explain the failure in otolith formation in *dlx3b/4b* mutants. Subsequent gene inactivation studies of *fam20cb* will be required to address its function in detail and might be relevant for the understanding of vestibular disorders.

## MATERIALS AND METHODS

### Ethical statement

Fish were kept according to FELASA guidelines (Aleström et al., 2020). All animal experiments were conducted according to the guidelines and under supervision of the Regierungspräsidium Dresden (permit: TVV 21/2018). All efforts were made to minimize animal suffering and the number of animals used.

### Zebrafish husbandry and lines

Zebrafish were kept and bred according to standard procedures (Brand et al., 2002; Westerfield, 2000). Zebrafish embryos were obtained by natural spawnings of adult fish and staged according to hours post fertilization (hpf) or standard criteria (Kimmel et al., 1995). The wild-type line used was AB. The transgenic line Gt(pax8:DsRedx) has been described previously (Ikenaga et al., 2011). Specifically, heterozygous animals were used throughout the experiments which are indistinguishable from non-transgenic wild-type siblings with respect to their phenotype. The deletion removing the loci of *dlx3b* and *dlx4b* (*Df(Chr12:dlx3b, dlx4b)tud70*) including the genotyping protocol has been described (Schwarzer et al., 2017).

### Morpholino injections

Zebrafish morpholino oligomers (MOs) were obtained from Gene Tools, Inc. MOs for *dlx3b* (5′-ATGTCGGTCCACTCATCCTTAATAA-3′), *dlx4b* (5'-GCCCGATGATGGTCTGAGTGCTGC-3′) and *atoh1b* (5′-TCATTGCTTGTGTAGAAATGCATAT-3′) were described previously (Hans et al., 2013; Millimaki et al., 2007). About 1-3 nl of MO-solution was injected into the cytoplasm of one-cell-stage embryos.

### Tissue dissociation and fluorescence-activated cell sorting (FACS)

Tissue dissociation was conducted as described previously (Manoli and Driever, 2012). Briefly, embryos were grown up to late OEPD stages corresponding to the 6-9-somite stage (12-13.5 hpf). Subsequently, embryos were removed from their chorions by pronase treatment (Westerfield, 2000), followed by deyolking at 4°C in 0.5% Ginzburg-Ringer without CaCl_2_. Dissociation was conducted in trypsin-EDTA on ice. When embryos were completely dissociated, the reaction was stopped by adding Hi-FBS. The cells were pelleted, washed with PBS, resuspended in PBS and passed through a 40μM mesh filter prior to cell sorting. FACS was performed using an Aria II cell sorter (BD Biosciences). Forward and side scatter were used to gate for live, single cells, out of which DsRed-positive cells were sorted and collected (Fig. S1). Flow cytometry data were analyzed using BD FACSDiva software.

### RNA isolation from FAC-sorted cells

RNA isolation from sorted cells was performed using the Total RNA purification Micro Kit (Norgen Biotek) following the manufacturer's protocol with slight modifications: prior to isolation, 200 μl aliquots of the Buffer RL were desiccated at 60°C for 1 h using an Eppendorf Concentrator plus with the V-AQ mode and stored at room temperature. The exact volume of the sorted samples was evaluated. 1% of the sample volume β-Mercaptoethanol was added and the whole suspension was added to the desiccated RL Buffer. The salts were dissolved using a vortex mixer. Next, the volume was measured and 100% EtOH were added at a ratio of 1:1.75, mixed and the suspension was put on a spin column. The sample was centrifuged 1 min at 14.000×***g*** and washed twice using 400 μl of wash solution A, discarding the flow through and centrifuged 1 min at 14.000×***g***. Finally, the column was spin-dried for 2 min, placed in a new tube and eluted using 20 μl RNase-free H_2_O. Sample collection was done by centrifugation at 14.000×***g***. 1.5 μl were used for RNA quality analysis using an Agilent Bioanalyzer. All samples were analyzed prior to RNA sequencing by the DNA Microarray Facility of the MPI-CBG Dresden and stored at −80°C until sequencing was performed.

### Next generation sequencing

RNA sequencing was performed on three biological replicates for each condition. RNA sequencing was based on Smart-seq2 sensitive full-length transcriptome profiling and modified from (Picelli et al., 2013). For reverse transcription, 2 μl of a primer mix was added. RNA was then denatured for 3 min at 72°C and the reaction was performed at 42°C for 90 min after filling up to 10 μl with reverse transcription buffer mix. The reverse transcriptase was inactivated at 70°C for 15 min and the cDNA was amplified using Kapa HiFi HotStart Readymix (Roche, #KK2601) at a final 1× concentration and 0.1 μM UP-primer (UP-primer: AAGCAGTGGTATCAACGCAGAGT). The amplified cDNA was then purified using 1× volume of hydrophobic Sera-Mag SpeedBeads (GE Healthcare, #11829912) and DNA was eluted in 12 μl nuclease-free water. The concentration of the samples was measured with a Tecan plate reader Infinite 200 pro, in 384 well black flat-bottom, low-volume plates (Corning), using AccuBlue Broad range chemistry (Biotium, #31007). For library preparation, 700 pg cDNA in 2 μl were mixed with 0.5 μl Tagment DNA Enzyme, 2.5 μl Tagment DNA Buffer (Nextera, Illumina, #20034197) and tagmented at 55°C for 5 min. Subsequently, Illumina indices were added during PCR with 1x concentrated KAPA Hifi HotStart Ready Mix (Roche, #KK2601) and 0.7 μM dual indexing primers. After PCR, libraries were quantified with AccuBlue Broad range chemistry, equimolarly pooled and purified twice with 1x volume Sera-Mag SpeedBeads. This was followed by Illumina sequencing on a Nextseq500, resulting in ∼26-34 million single end reads per library. After sequencing, FastQC (http://www.bioinformatics.babraham.ac.uk/) was used to perform a basic quality control on the resulting reads. Alignment of the reads to the zebrafish reference (GRCz11) was performed with GSNAP (2018-07-04) (Wu and Nacu, 2010) and Ensembl gene annotation version 92 helped to detect splice sites. Afterwards, library diversity was assessed by redundancy investigation in the reads. The uniquely aligned reads were counted with featureCounts (1.6.2) (Liao et al., 2013) and the support of the same Ensembl annotation file. Normalization of the raw read counts, based on the library size and testing for differential expression between 1 dpl and unlesioned samples was performed with the DES Eqn (1.18.1) R package (Benjamini and Hochberg, 1995; Love et al., 2014). We used a multi-factor design within the differential expression analysis, to control for the detected clutch effect. Genes which have an adjusted *P* value (padj) <0.1 were considered as differentially expressed.

### Gene ontology analysis

For gene ontology analysis, the tool GOrilla (http://cbl-gorilla.cs.technion.ac.il/) was employed. To do so, the 3.015 differentially expressed genes (DEGs) obtained from control versus Dlx3b/4b-depleted samples with a false discovery rate of 10% were copied into the input panel of the tool which was last updated on 18 August 2018. *Danio rerio* (Zebrafish) was chosen as the analysis organism, and a process ontology was selected. Parameters were set as default as suggested by the tool. The system recognized 2209 genes out of the 3015 gene terms entered. Only 1988 of these genes were associated with a GO term. ‘*P*-value’ is the enrichment *P*-value computed according to the mHG or HG model. This *P*-value is not corrected for multiple testing of 4387 GO terms. ‘FDR q-value’ is the correction of the above *P*-value for multiple testing using the Benjamini and Hochberg (1995) method. Namely, for the i^th^ term (ranked according to *P*-value) the FDR q-value is (*P*-value *number of GO terms) / i. Enrichment=(b/n) / (B/N), in which N, B, n, and b are defined as follows: N: total number of genes. B: total number of genes associated with a specific GO term. *N*: number of genes in the top of the user's input list or in the target set when appropriate. b: number of genes in the intersection.

### *In situ* hybridization

Published cDNA probes for the following genes were used: *myo7aa* (Ernest et al., 2000); *atoh1a* (Itoh and Chitnis, 2001); *atoh1b* (Adolf et al., 2004); *has3* (Geng et al., 2013); *robo4* (Bedell et al., 2005); *pcdh7b* (Blevins et al., 2011); *rsph9* (Sedykh et al., 2016); *sox9b* (Chiang et al., 2001); *irx4b* (Lecaudey et al., 2005); *gfi1ab* (Dufourcq et al., 2004) and *agr2* (Shih et al., 2007). Gene fragments were cloned and used as probes for the following genes (see Supplementary Information): *ptchd3a*, *stc2a*, *si:ch211-137a8.2*, *fam20cb*, *ccdc103*, *klhl14*, *mcf2lb*, *zgc:194210*, *foxj1b*, *mns1*,*ulk1a*, *cdr2l*, *zgc:158291*, *si:dkey222f2.1* and *cxcl14*. Probe synthesis and *in situ* hybridization were performed essentially as previously described (Westerfield, 2000). Gene fragments of *ptchd3a*, *stc2a*, *si:ch211-137a8.2*, *fam20cb*, *ccdc103*, *klhl14*, *mcf2lb*, *zgc:194210*, *foxj1b*, *mns1*,*ulk1a*, *cdr2l*, *zgc:158291*, *si:dkey222f2.1* and *cxcl14* were cloned into Topo pCRII (ThermoFisher Scientific) from genomic DNA using standard PCR with the primers provided in Table S5. Sequencing confirmed the identity of the respective gene fragment, which was subsequently used as probe.

### Heat treatments

For heat treatments, embryos, still in their chorions, were transferred into fresh Petri dishes. After removal of excess embryo medium, pre-heated 42°C warm embryo medium was added and the Petri dishes were kept for 30 min in a 37°C incubator before they returned to a 28.5°C incubator.

### Generation of an *atoh1b* mutant line

Cas9 mRNA and *atoh1b* gRNAs were synthesized as recently described (Jao et al., 2013; Shah et al., 2015). Briefly, Cas9 mRNA was synthesized by *in vitro* transcription using T3 mMESSAGE mMACHINE kit (Ambion). gRNAs were generated and purified using the MEGAshortscript T7 and phenol/chloroform, respectively. Sequences of the genomic target sites and oligonucleotides for making gRNAs are as follows.


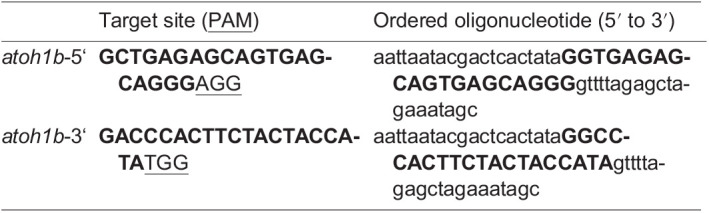


For germ line transformation, Cas9 mRNA and gRNAs were co-injected into fertilized eggs, embryos were raised to adulthood, crossed to AB wild-type fish and the resulting F1 embryos were screened by PCR. To detect the deletion in the *atoh1b* locus, the primers atoh1b-1f 5′- AAACTGTGATCATCCTGCGGAAAGC-3′ and atoh1b-rev 5′- CCTAACTTTACCCTAATTACCCTAGTGAAGCC-3′ were used generating an amplicon of 822 base pairs (bp) in the presence of the deletion allele. In total, 50 animals were screened and one founder carrying the *atoh1b* deletion in the germline was identified. For subsequent genotyping, the primer atoh1b-2f 5′- GTCGACTTGTCATGTTTAAGGCGATGG-3′ was added amplifying an 862-bp fragment in the presence of wild-type DNA. Genotyping of embryos demonstrated the exact concordance between homozygous mutant genotype and observed phenotype. At least 12 mutant embryos from three separate clutches were analyzed for each experiment.

### Generation of the *Tg(hsp70l:mCherry-T2a-atoh1b)* transgenic line

To create the pTol hsp70l:mCherry-T2a-atoh1b plasmid, the coding sequence of atoh1b was PCR amplified from genomic DNA with primers atoh1b-orf-for (5′-TATAgctagcACTGCAAAAACGAAGCTTTTGCATTGGAC-3′) and atoh1b-orf-rev (5′-TATAagatctTCAGCGTCCTCCAGTGTGTCC-3′) flanked by the unique restriction sites *Nhe*I and *BglI*I, respectively. After digestion, the PCR product was cloned into the vector pTol hsp70l:mCherry-T2a-CreER^T2^ (Hans et al., 2011) replacing the CreER^T2^ coding sequence. For germ line transformation, plasmid DNA and transposase mRNA were injected into fertilized eggs (F0), injected embryos were raised to adulthood and crossed to AB wild-type fish as previously described (Abe et al., 2004). To identify transgenic carriers, undechorionated F1 embryos at 24 hpf were heat treated, examined under a fluorescent microscope after a 4 h waiting period and mCherry-positive embryos were raised. This way, five independent F0s were identified and one allele was chosen to establish the line.

### Image acquisition and processing

Images were taken with a Zeiss Axio Imager Z1 or an Olympus MVX microscope equipped with Olympus DP80 digital camera and the cellSens Dimension imaging software. Images were processed using Adobe Photoshop CC2015. Figures were assembled using Adobe Illustrator CC2015. Scatter plots of genes including standard deviation were assembled using graph pad prism software based on the read counts obtained from RNAseq.

## Supplementary Material

10.1242/biolopen.059911_sup1 Supplementary information
