# Supplementary material for: Transcriptome analysis reveals an Atoh1b-dependent gene set downstream of Dlx3b/4b during early inner ear development in zebrafish
Source: Biol Open. 2023 Jun 5;12(6):bio059911. doi: 10.1242/bio.059911 (PMC10261724; doi:10.1242/bio.059911)
Supplement: Supplementary information [file biolopen-12-059911-s1.pdf]

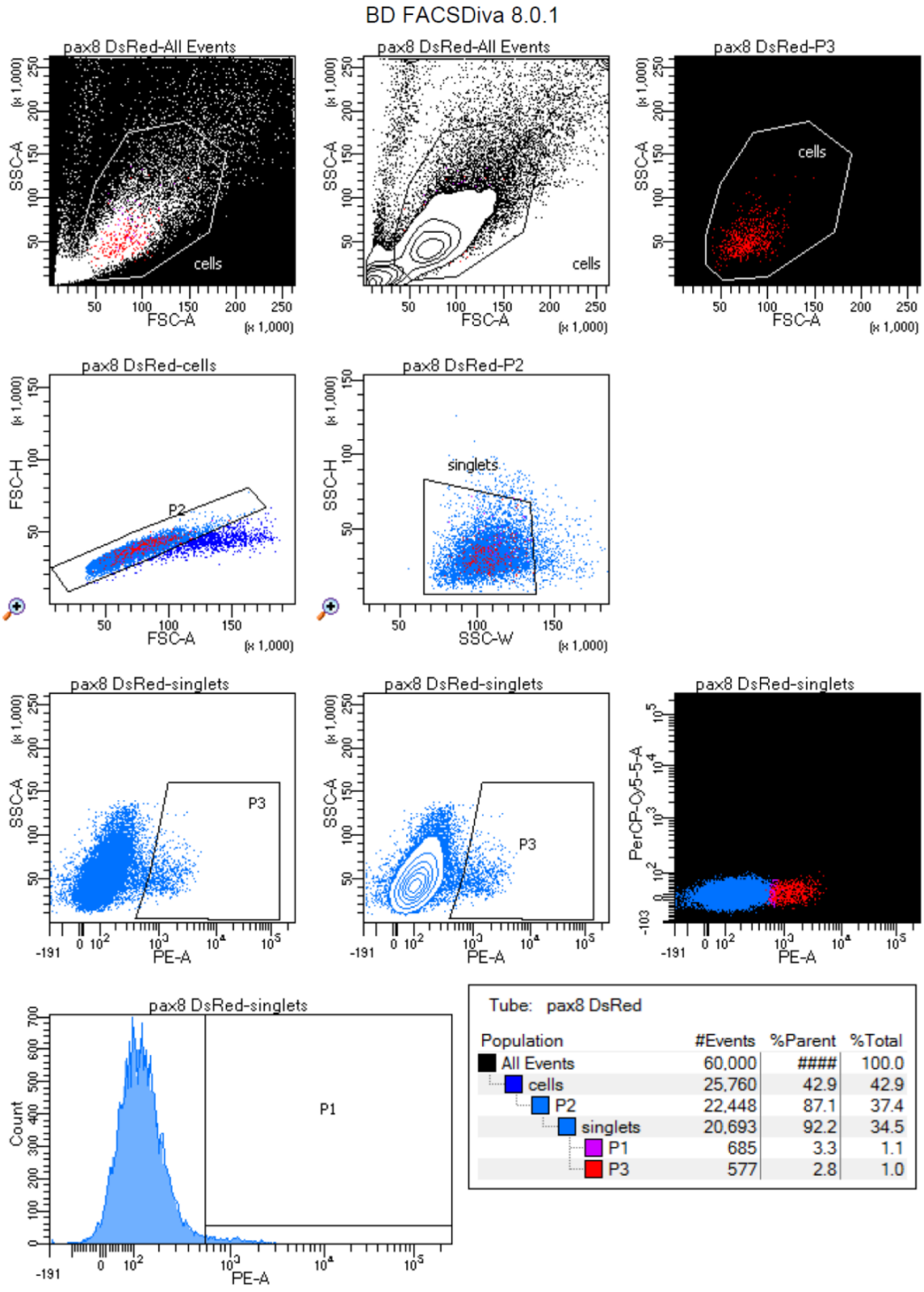

Fig. S1. FACS plot of *pax8*:DsRed-positive cells and their respective events during gating.

Biology Open • Supplementary information

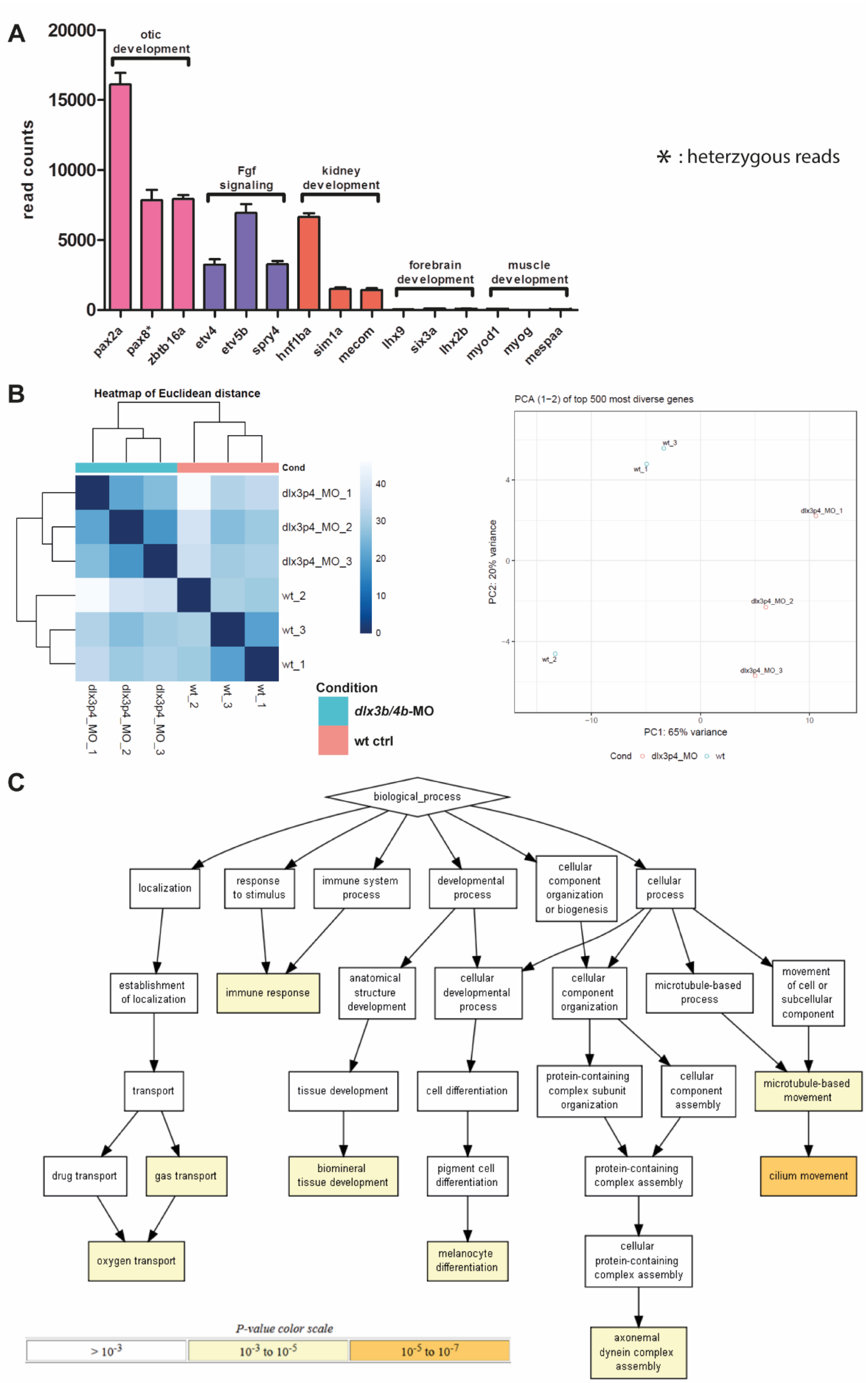

Biology Open • Supplementary information

Fig. S2. (A) Downstream genes of the Fgf signaling pathway as well as genes associated with early inner ear and kidney but not forebrain and muscle development are highly enriched in the *pax8*:DsRed-sorted cells. (B) Using Euclidean distance and principal component analysis the three biological control and Dlx3b/4b-depleted samples cluster together. (C) Gene ontology (GO) analysis using the GOrilla gene ontology analysis tool (Eden et al., 2009).

A

GO term	Description	P-value	FDR q-value	Enrichment
GO:0034969	histone arginine methylation	9.51E-04	2.74E-02	12.93
GO:0003343	septum transversum development	9.51E-04	2.73E-02	12.93
GO:0003342	proepicardium development	9.51E-04	2.73E-02	12.93
GO:0030859	polarized epithelial cell differentiation	1.44E-03	3.86E-02	11.09
GO:0010002	cardioblast differentiation	3.88E-04	1.26E-02	10.78
GO:1905770	regulation of mesodermal cell differentiation	3.88E-04	1.26E-02	10.78
GO:0042661	regulation of mesodermal cell fate specification	3.88E-04	1.25E-02	10.78
GO:0036342	post-anal tail morphogenesis	1.88E-07	1.46E-05	10.16
GO:0001710	mesodermal cell fate commitment	5.58E-04	1.70E-02	9.70
GO:0048385	regulation of retinoic acid receptor signaling pathway	1.06E-03	2.97E-02	8.08

B

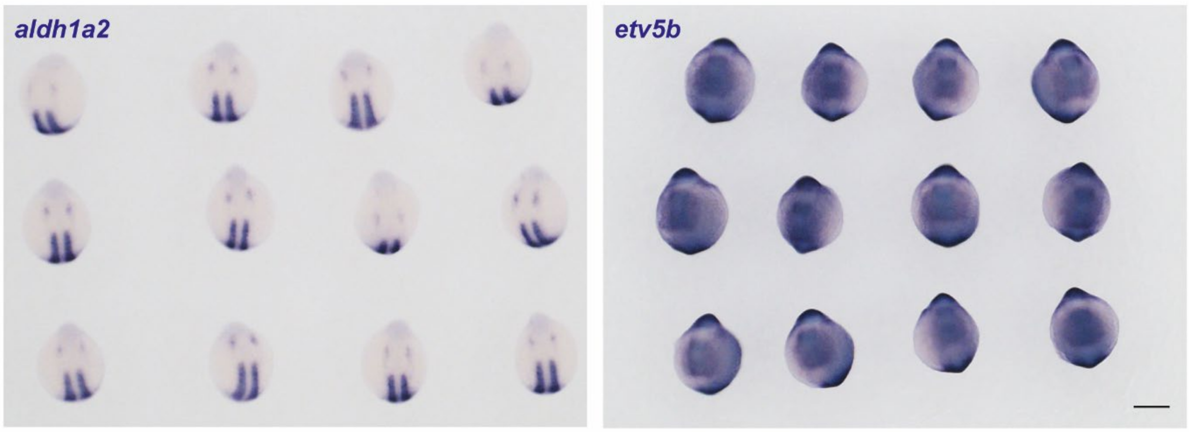

Fig. S3. (A) Gene ontology (GO) analysis of upregulated DEGs only reveals mostly GO terms associated with mesodermal tissue development. (B) Analysis of the two upregulated genes, *aldh1a2* and *etv5b*, using *in situ* hybridization on embryos at late OEPD stages (6-9-somites) obtained from incrosses of heterozygous animals carrying the deletion allele of *dlx3b/4b* (Schwarzer et al., 2017). No striking differences in expression were observed. Dorsal views, anterior to the top. Scale bar: 500 µm.

Biology Open • Supplementary information

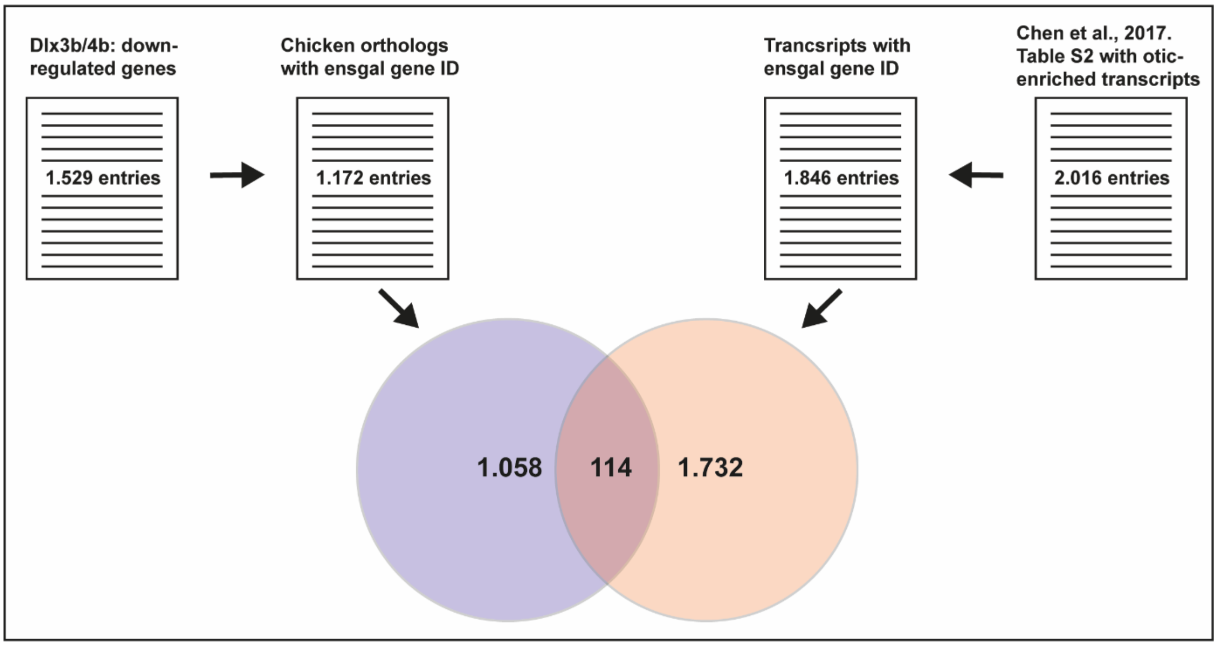

Fig. S4. (A) Comparison of the genes downregulated after Dlx3b/4b depletion and the transcripts expressed within the chicken OEPD (Table S2 from (Chen et al., 2017)). The list of downregulated genes following Dlx3b/4b depletion contained 1.529 genes and resulted in the identification of 1.172 chicken orthologs with an ensgal gene ID. The initial list from Chen et al., initially contained 2.016 genes in total with 1.846 transcripts owing an ensgal gene ID. Comparison revealed 114 genes to be expressed in the OEPD of chicken and zebrafish (see Table S3).

Biology Open • Supplementary information

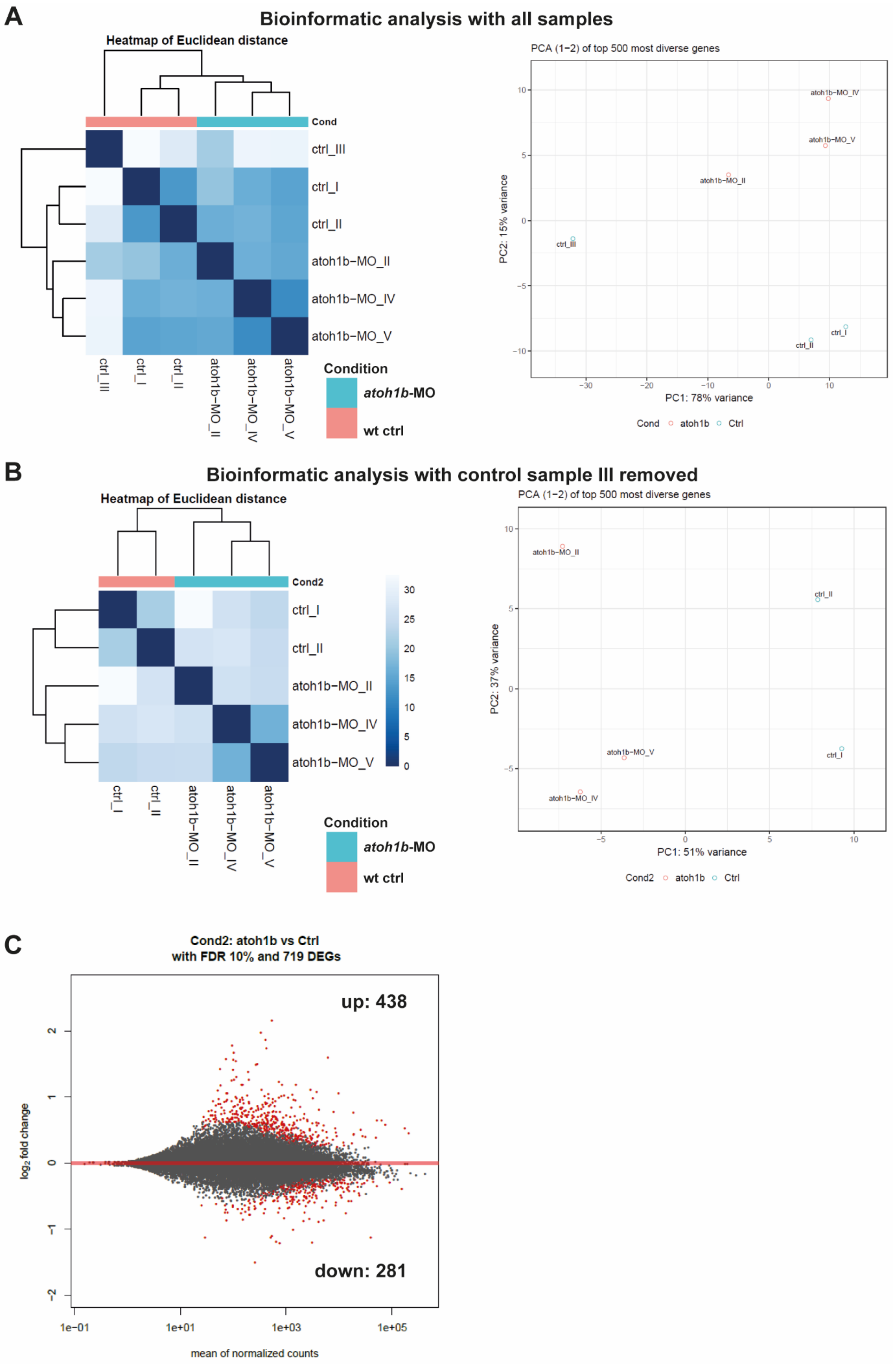

Fig. S5. (A) Euclidean distance and principal component analysis clustered the three biological Atoh1b-depleted samples cluster but not the three control samples. (B) Euclidean distance and principal component analysis using three biological Atoh1b-depleted and only two control samples. (C) Comparison of control versus Atoh1b-depleted samples with a false discovery rate (FDR) of 10 % revealed 719 differentially expressed genes.

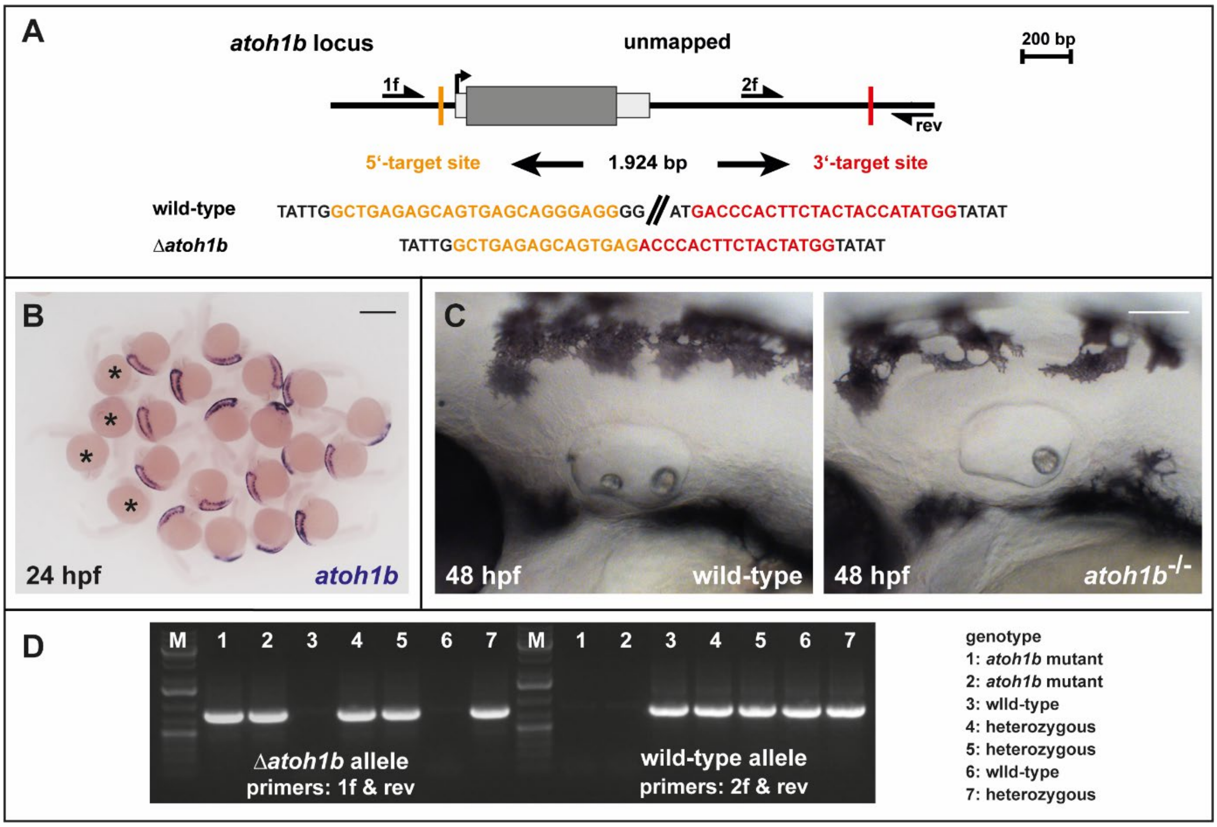

Fig. S6. Generation of an *atoh1b* null allele. (A) Scheme of the *atoh1b* locus at an unmapped chromosome. The transcriptional start site is indicated with an arrow. Exon sequences with translated and untranslated regions are represented in dark and light grey, respectively. Positions of the CRISPR/Cas9 target sequences, separated by 1.924 bp, and their sequences in the wild-type and the *atoh1b* deletion allele are indicated in orange and red. Primers used for genotyping (1f, 2f and rev) are shown as half arrows. (B) *in situ* hybridization of *atoh1b* at 24 hours post fertilization (hpf) reveals loss of *atoh1b* in a quarter of embryos (asterisk) obtained from an *atoh1b* heterozygote incross. (C) Lateral views of live images of wild-type control embryos and *atoh1b* mutants. Lateral views are seen with anterior to the left. Scale bars: (B) 500 µm (C) 75 µm. (D) PCR using primers 1f and rev or 2f and rev reveals the presence of the *atoh1b* deletion (822 bp) and wild-type allele (862 bp), respectively. Genotyping of two embryos with otolith phenotype (#1, #2) and five embryos with wild-type morphology (#3-7) confirm absence of the wild-type allele in the former. M indicates marker for molecular size standard.

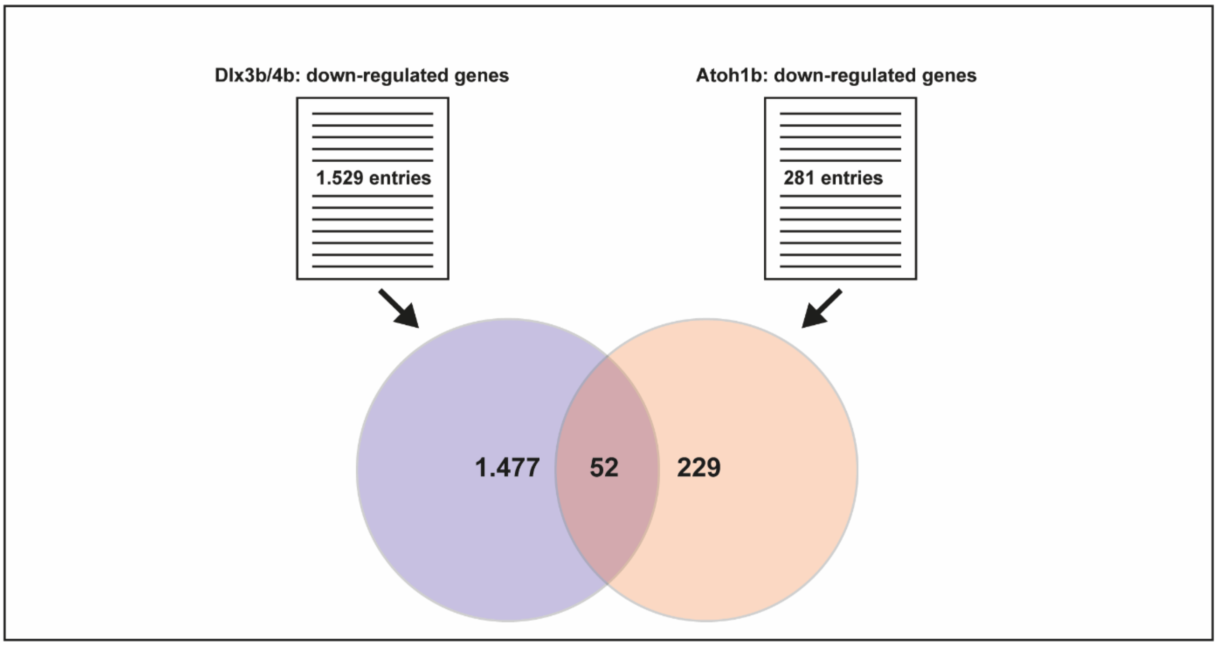

Fig. S7. Comparison of 1.529 genes downregulated after Dlx3b/4b depletion with 281 genes downregulated after Atoh1b depletion reveals 52 genes downregulated in both conditions (see Table S5).

Biology Open • Supplementary information

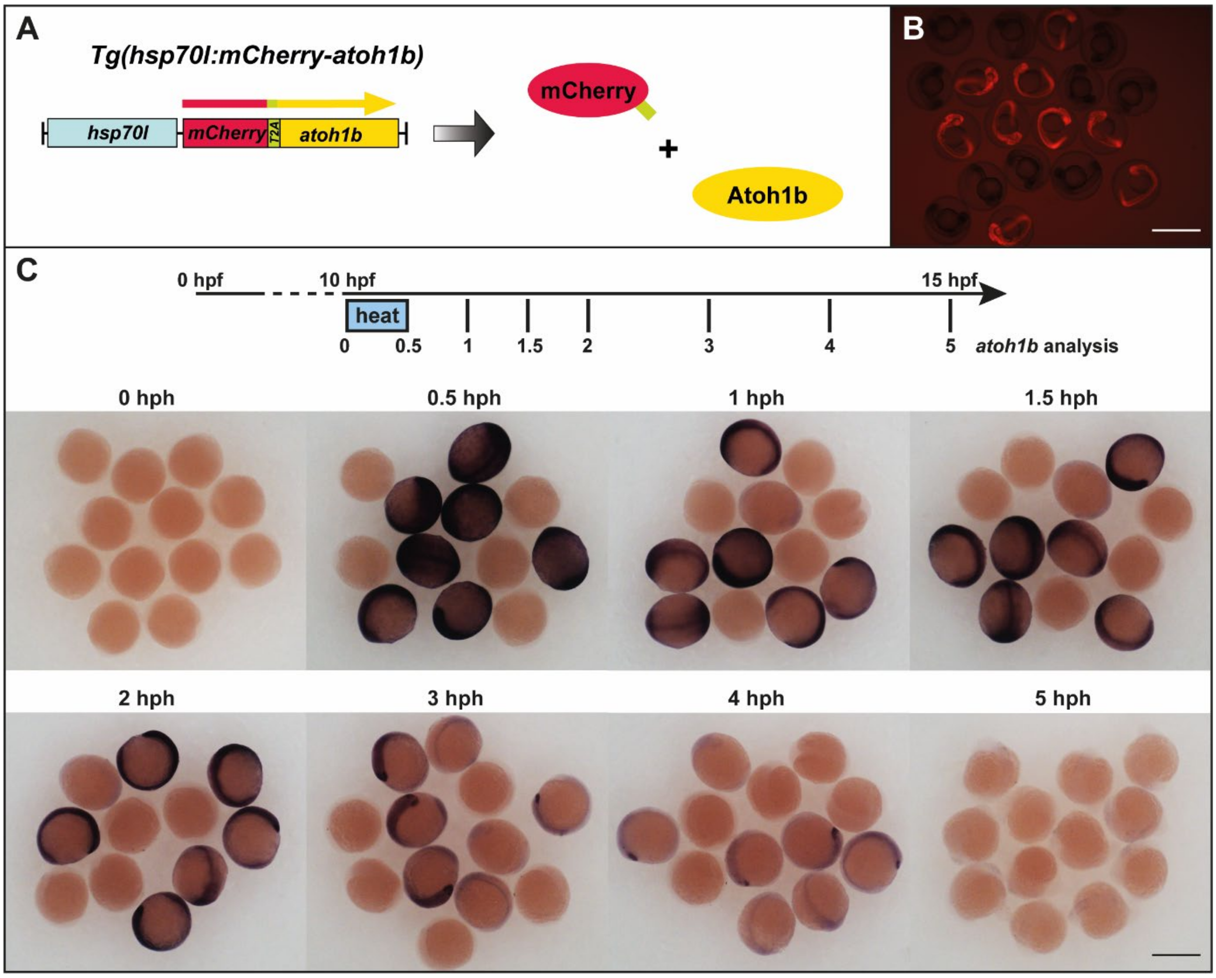

Fig. S8. Generation of the *Tg(hsp70l:mCherry-T2a-atoh1b)* transgenic line. (A) Scheme of the *Tg(hsp70l:mCherry-T2A-atoh1b)* construct expressing a single open reading frame coding for mCherry (red) and Atoh1b (yellow) separated by a viral T2A peptide sequence (green) under the control of a zebrafish heat shock-inducible *hsp70l* promoter (blue). After translation, the viral T2A peptide cleavage leads to the production of non-fused mCherry and Atoh1b proteins. (B) Identification of transgenic animals expressing mCherry at 28 hours post fertilization (hpf) after a heat treatment at 24 hpf. (C) Following a 30 minute heat treatment at the end of gastrulation (10 hpf), strong and ubiquitous expression of *atoh1b* can be observed in transgenic embryos up to 4 post hours post heat treatment (hph). Ectopic *atoh1b* is gradually lost and heat-treated transgenic embryos cannot be discriminated any longer based on *atoh1b* expression from non-transgenic siblings. Scale bar: (B) 1000 µm, (C) 500 µm.

Biology Open • Supplementary information

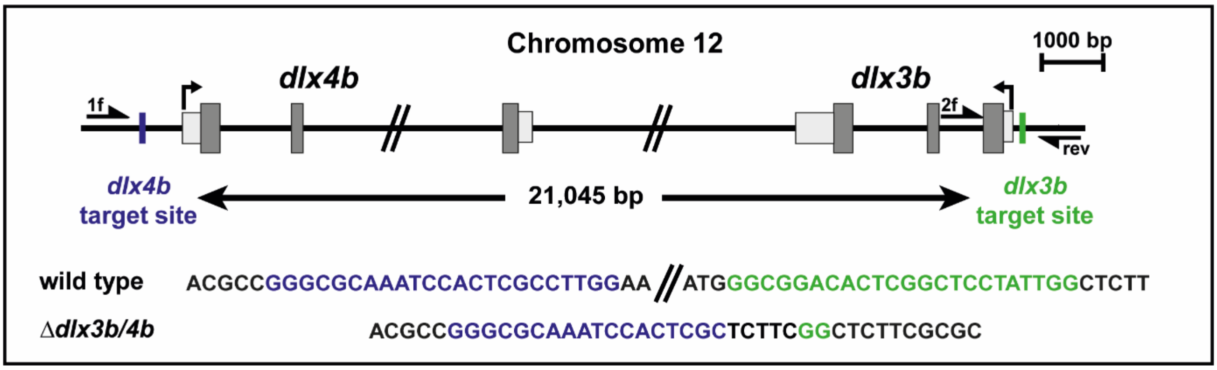

Fig. S9. Scheme of the *dlx3b/4b* bigene cluster at chromosome 12. Transcriptional start sites are indicated with arrows. Exon sequences with translated and untranslated regions are represented in dark and light grey, respectively. Positions of the CRISPR/Cas9 target sequences, separated by 21.045 bp, and their sequences in the wild-type and the *dlx3b/4b* deletion allele are indicated in blue and green. The three primers 1f, 2f and rev used for genotyping are shown as half arrows. Primer combination 1f and rev detects the *dlx3b/4b* deletion allele with a 618 base pair amplicon. Primer combination 2f and rev detects the wild-type allele with a 473 base pair amplicon. Figure adapted from (Schwarzer et al., 2017).

Biology Open • Supplementary information

Table S1. Read counts of all genes in wild-type and *dlx3b/4b*-morpholino injected samples.

Click here to download Table S1

Table S2. Differentially expressed genes from wild-type compared to Dlx3b/4b-depleted samples with a false discovery rate of 10 %.

Click here to download Table S2

Table S3. Comparison of otic-enriched transcripts identified in the chick OEPD provided in table S2 from Chen et al., 2017 and our gene set of downregulated DEGs.

Click here to download Table S3

Table S4. Differentially expressed genes from wild-type compared to Atoh1b-depleted samples with a false discovery rate of 10 %.

Click here to download Table S4

Table S5. Genes downregulated in Dlx3b/4b-and Atoh1b-depleted samples.

Click here to download Table S5

Table S6. Primers used to clone gene fragments of genes shown in this study.

Click here to download Table S6

Biology Open • Supplementary information
